# The Subcortical-Allocortical- Neocortical *continuum* for the Emergence and Morphological Heterogeneity of Pyramidal Neurons in the Human Brain

**DOI:** 10.3389/fnsyn.2021.616607

**Published:** 2021-03-11

**Authors:** Alberto A. Rasia-Filho, Kétlyn T. Knak Guerra, Carlos Escobar Vásquez, Aline Dall’Oglio, Roman Reberger, Cláudio R. Jung, Maria Elisa Calcagnotto

**Affiliations:** ^1^Department of Basic Sciences/Physiology and Graduate Program in Biosciences, Universidade Federal de Ciências da Saúde de Porto Alegre, Porto Alegre, Brazil; ^2^Graduate Program in Neuroscience, Universidade Federal do Rio Grande do Sul, Porto Alegre, Brazil; ^3^Medical Engineering Program, Friedrich-Alexander-Universität Erlangen-Nürnberg, Erlangen, Germany; ^4^Institute of Informatics, Universidade Federal do Rio Grande do Sul, Porto Alegre, Brazil; ^5^Neurophysiology and Neurochemistry of Neuronal Excitability and Synaptic Plasticity Laboratory, Department of Biochemistry and Biochemistry Graduate Program, Universidade Federal do Rio Grande do Sul, Porto Alegre, Brazil

**Keywords:** amygdaloid complex, hippocampus, cerebral cortex, human dendritic spines, 3D reconstruction, Alzheimer’s disease, temporal lobe epilepsy

## Abstract

Human cortical and subcortical areas integrate emotion, memory, and cognition when interpreting various environmental stimuli for the elaboration of complex, evolved social behaviors. Pyramidal neurons occur in developed phylogenetic areas advancing along with the allocortex to represent 70–85% of the neocortical gray matter. Here, we illustrate and discuss morphological features of heterogeneous spiny pyramidal neurons emerging from specific amygdaloid nuclei, in CA3 and CA1 hippocampal regions, and in neocortical layers II/III and V of the anterolateral temporal lobe in humans. Three-dimensional images of Golgi-impregnated neurons were obtained using an algorithm for the visualization of the cell body, dendritic length, branching pattern, and pleomorphic dendritic spines, which are specialized plastic postsynaptic units for most excitatory inputs. We demonstrate the emergence and development of human pyramidal neurons in the cortical and basomedial (but not the medial, MeA) nuclei of the amygdala with cells showing a triangular cell body shape, basal branched dendrites, and a short apical shaft with proximal ramifications as “pyramidal-like” neurons. Basomedial neurons also have a long and distally ramified apical dendrite not oriented to the pial surface. These neurons are at the beginning of the allocortex and the limbic lobe. “Pyramidal-like” to “classic” pyramidal neurons with laminar organization advance from the CA3 to the CA1 hippocampal regions. These cells have basal and apical dendrites with specific receptive synaptic domains and several spines. Neocortical pyramidal neurons in layers II/III and V display heterogeneous dendritic branching patterns adapted to the space available and the afferent inputs of each brain area. Dendritic spines vary in their distribution, density, shapes, and sizes (classified as stubby/wide, thin, mushroom-like, ramified, transitional forms, “atypical” or complex forms, such as thorny excrescences in the MeA and CA3 hippocampal region). Spines were found isolated or intermingled, with evident particularities (e.g., an extraordinary density in long, deep CA1 pyramidal neurons), and some showing a spinule. We describe spiny pyramidal neurons considerably improving the connectional and processing complexity of the brain circuits. On the other hand, these cells have some vulnerabilities, as found in neurodegenerative Alzheimer’s disease and in temporal lobe epilepsy.

“*… the cerebral cortex is similar to a garden filled with trees, the pyramidal cells, which, thanks to intelligent culture, can multiply their branches, sending their roots deeper and producing more and more varied and exquisite flowers and fruits.*” (Ramón y Cajal, 1894a)

## Introduction

Ramón y Cajal (1894b) described cortical pyramidal neurons (or “psychic cells”) as “progressively larger and more complex in ascending the animal scale… to assume that at least part of its increased functional role is a result of increased morphological complexity… In descending the vertebrate ladder, the shape of the psychic cell becomes simpler, with its length and volume decreasing in parallel… differences are in microscopic form and the relative volume of particular components used in (brain) construction.” In humans, pyramidal neurons are found in forebrain structures (Ramón y Cajal, 1894b, Ramón y Cajal, 1909–1911), but not in the striatum, the cerebellum, the brainstem, or in the spinal cord (Spruston, 2008). These cells develop in the “anatomic limbic system” or “greater limbic lobe” (Miller and Vogt, 1995; Wyss and van Groen, 1995; Gloor, 1997; Heimer et al., 2008) and are found in the heterogeneous allocortex and neocortex layers (i.e., except in cortical layer I, from layers II to VI and their subdivisions; Miller and Vogt, 1995; Andersen et al., 2007; DeFelipe, 2011), accounting for approximately 70–85% of all cells in the cerebral gray matter (Nieuwenhuys, 1994; DeFelipe, 2011; Kolb and Whishaw, 2015).

Pyramidal neurons have been studied using complementary techniques, from the Nissl (or thionine) staining and the Golgi silver impregnation procedure to different approaches for intracellular microinjection of fluorescent dyes, serial sections for ultrastructural connectional and neurochemical profiles, *in vitro* and *in vivo* electrophysiological recordings, computational and *in silico* models (Ramón y Cajal, 1909–1911; Lorente de Nó, 1934; Szentágothai, 1978; Mountcastle, 1979; Braak, 1980; Peters and Jones, 1984; Sims and Williams, 1990; Peters et al., 1991; McCormick et al., 1993; Segev et al., 1995; Somogyi et al., 1998; Valverde et al., 2002; Andersen et al., 2007; Spruston, 2008; Larriva-Sahd, 2010; Ramaswamy and Markram, 2015; Eyal et al., 2018; Soltesz and Losonczy, 2018; Cembrowski and Spruston, 2019; Oruro et al., 2019; Benavides-Piccione et al., 2020). For example, the Golgi method adapted for formalin-fixed human brain and light microscopy provides images of pyramidal dendrites and spine shapes from different cortical and subcortical regions (Dall’Oglio et al., 2010; Reberger et al., 2018; Vásquez et al., 2018; Correa-Júnior et al., 2020). This can eventually add fundamental data to identify the brain cellular components and their connectivity toward physiology and behavior, as well as for modeling and theory approaches on neural structure and integrated functions of human brain areas (see a current discussion in Zeng, 2020).

The “typical” or “classical” morphological attributes of an adult spiny pyramidal neuron include: (1) a triangular/conical soma; (2) basal dendrites with opposing origins from the base of the perikaryon and closely ramified branches extending radially outward; (3) an apical dendrite arising from the apex of the cell body with some collaterals branches but maintaining a straight course until the terminal ramification near the cortical surface; (4) an heterogeneous distribution of spines from proximal to distal dendrites; and (5) an axon that descends toward the white matter (Feldman, 1984; Peters and Jones, 1984; for further information see Nieuwenhuys, 1994). These features typically refer to thick-tufted pyramidal neurons in the neocortical internal pyramidal layer V of various species (e.g., rats, mice, monkeys, and humans; Feldman, 1984; Peters and Jones, 1984; Ledergerber and Larkum, 2010; Ramaswamy and Markram, 2015; Wang et al., 2018). The study of pyramidal neurons is vast, and we still do not have a complete picture of the integrated functions of these abundant cells in the most complex neural processing, such as consciousness, cognition, abstract thinking, creativity, and social emotions (see relevant data in Anderson et al., 2009; DeFelipe, 2011; Marín-Padilla, 2014; Ramaswamy and Markram, 2015; Cembrowski and Spruston, 2019 and references therein). Morphology is a crucial step to proceed on this endeavor (Ramón y Cajal, 1894b; DeFelipe, 2011).

Here, we illustrate and discuss the morphological findings of three-dimensional (3D) reconstruction of heterogeneous pyramidal neurons with pleomorphic dendritic spines in the anatomical and functional subcortical-allocortical-neocortical *continuum* in the human brain (from adult males; samples and methodological procedures are described in Dall’Oglio et al., 2010, 2013, 2015; Reberger et al., 2018; Vásquez et al., 2018). Our aim is not to exhaustively elaborate the data available in the literature. Additional references can be found in the articles cited here. Instead, we would like to highlight and instigate further 3D morphological studies on the emergence and development of human pyramidal neurons, including the features of dendritic spine number and shapes, as essential steps for understanding the integrative capacities of these neurons in distinct, functionally specialized brain areas (Spruston, 2008; Luebke, 2017; Soltesz and Losonczy, 2018; Cembrowski and Spruston, 2019; Benavides-Piccione et al., 2020). These human data encourage further efforts on elaborating the cell heterogeneity and synaptic processing in dendritic domains and spines of pyramidal cells settled from specific amygdaloid nuclei to neocortical areas in both normal and pathological conditions. In this regard, pyramidal neurons show vulnerabilities and involvement in Alzheimer’s disease (AD) and epilepsy, as described below.

## The Morphological Heterogeneity of Pyramidal Neurons

### “Typical” Pyramidal and “Pyramidal-Like” Neurons

The classification of a neuron as a pyramidal cell type includes morphological features that show considerable diversity in each brain area within and across species (Ramón y Cajal, 1894b; Feldman, 1984; Nieuwenhuys, 1994; Spruston, 2008; Ledergerber and Larkum, 2010; Bianchi et al., 2013; Luebke, 2017; Soltesz and Losonczy, 2018; Cembrowski and Spruston, 2019; Gouwens et al., 2019; Benavides-Piccione et al., 2020). From a morphological standpoint, heterogeneous pyramidal neurons can have a small to large cell body with triangular, spherical, ovoid, rhomboidal, and irregular forms, basal dendrites with varied branching pattern and radial extension, and an apical dendrite with different terminal tuft aspect (Feldman, 1984). Large thick-tufted pyramidal neurons in neocortical layer V have basal and apical dendrites whose length would integrate afferent connections across different layers. However, there are variations in these cells for their basal ramification, apical bifurcation, and tapering as a horizontal tuft in the superficial cortical layers (Morishima and Kawaguchi, 2006; Wang et al., 2018). In the rat frontal cortex, two populations of layer V pyramidal neurons that project to the striatum differ in apical dendrite initial shaft diameter and the distal tuft area, length, and branch points in layer I (Morishima and Kawaguchi, 2006). That is, cells in the superficial layer V show tufted or slender apical dendrites, whereas cells in the deeper layer V have a reduced or absent apical tuft (Morishima and Kawaguchi, 2006). Furthermore, the apical dendrites of deeper layer VI pyramidal neurons may not reach layer I as a terminal tuft; rather, these dendrites taper at midcortical levels in the neocortex of rats and monkeys (Braak, 1980; Feldman, 1984; Ledergerber and Larkum, 2010).

“Typical” pyramidal neurons have a main apical dendritic shaft directed toward the pial surface of the neocortex. “Pyramidal-like” neurons show most features of a pyramidal shape (Gloor, 1997; Luis de la Iglesia and Lopez-Garcia, 1997) although they can have an apical dendrite branching close to the cell body and with different orientation in the neuropil (Vásquez et al., 2018). For example, the pyramidal-like neurons in the human cortical nucleus of the amygdaloid complex (posterior part, PCo) have a triangular cell body, two basal dendrites of a similar thickness, and one main thick dendrite emerging at the apex of the soma. The primary and short “apical” dendrite of these cells may not be directed to the nuclear external surface (Vásquez et al., 2018; shown below). Pyramidal-like neurons in the basolateral amygdaloid nuclei show three to five primary dendrites. One of them is at the somatic apex, it is longer than the others, and has no preferred spatial orientation (Braak and Braak, 1983; Gloor, 1997). These pyramidal-like neurons are not arranged in evident layers and are not oriented in parallel alongside one another (Gloor, 1997). Pyramidal-like neurons also show a pyramidal or piriform soma in the rat allocortex (subiculum), one thicker apical dendrite projecting across the molecular layer into the hippocampal fissure, and thinner basal projections into the alveus white matter (Mattia et al., 1997), or a more complex dendritic architecture in the *stratum oriens* of CA2 to CA1 regions of *Proechimys* (Scorza et al., 2011).

### “Modified Pyramids”

Lorente de Nó (1934) used the term “modified pyramids” for the main components of the “Ammon’s horn and fascia dentata” in man and monkey. Peters and Jones (1984) refer to modified pyramids when “there are other cells which are modified in form, but nonetheless are easily recognized as having pyramidal features” as those in the neocortical layer II (even with short, divaricated, or absent apical dendrites) or with a rather oval cell body, thin apical dendrite, and basal dendrites radiating out in all directions in neocortical layer IV. Modified pyramids refer to a great variety of cellular shapes (Braak, 1980). In the human isocortical multiform layer VI, modified pyramidal neurons “deviate substantially from stereotypical pyramidal cells” including cells with a short and thin apical dendrite, basal dendrites with different diameters and lengths, one thick basal dendrite extending in various directions or various dendrites generated from the lateral surfaces of the soma (Braak, 1980; Braak and Braak, 1985). In the piriform cortex, modified pyramids include bi-horn, spindle-, triangular-, and crescent-shaped cells (reviewed in Larriva-Sahd, 2010). “Inverted” pyramidal neurons in neocortical layer VI display an “apical” dendrite directed toward the white matter (Feldman, 1984; Steger et al., 2013). Other neurons were also considered variations (or specializations) of pyramidal neurons, such as the Meynert-Cajal cells in layer IVb of the primary visual cortex (Hof and Morrison, 1990), the Meynert neurons in layer Vb of the striate area (Braak, 1980), the large Betz cells in layer Vb of the primary motor cortex (Braak, 1980; Feldman, 1984), and the von Economo neurons (VENs) in the frontoinsular and anterior cingulate cortices, for example (Butti et al., 2013; Banovac et al., 2019; for VENs particularities see also Cauda et al., 2014; Yang et al., 2019; Correa-Júnior et al., 2020). Then, it is conceivable that the term “pyramidal neuron” might refer to a variety of shapes ranging from “classic” pyramidal, “pyramidal-like,” and “modified pyramids” with variable size, somatic shape, dendritic branching pattern, length, and orientation in the neuropil.

## Dendrites and Spines in Pyramidal Neurons

Besides conserved basic principles of mammalian brain development, evolution also produced significant quantitative and qualitative changes in cell number and shape along with circuit organization in cortical areas (Geschwind and Rakic, 2013; see also Herculano-Houzel et al., 2008; Herculano-Houzel, 2019). The connectivity of pyramidal dendrites in cortical multimodal areas, which receive a broad range of inputs at hierarchically higher association levels of integrative processing, show longer, more branched, and have more spines than in areas that process a specific modality of motor or primary sensory activity (Jacobs et al., 2001; Anderson et al., 2009; Kolb and Whishaw, 2015; Hrvoj-Mihic et al., 2017; González-Burgos et al., 2019). Moreover, cortical pyramidal neurons developed basal and apical dendritic domains with different synaptic receptive fields (Larriva-Sahd, 2002; Andersen et al., 2007; Spruston, 2008; Spruston et al., 2013; Larriva-Sahd, 2014). These dendritic segments can (1) compartmentalize signals and/or sum and organize synchronized transmission of information, both of which provide much more computational capabilities for the dynamic processing of information; (2) use passive and/or active membrane properties; (3) show anterograde and retrograde action potentials; and (4) depending on intrinsic membrane properties, impose refractory periods and/or a selective excitability of a specific segment depending on time and distance in the dendritic tree (Oakley et al., 2001; Andersen et al., 2007; Spruston, 2008; Spruston et al., 2013; Almog and Korngreen, 2014; Kastellakis et al., 2016).

The development of particularly specialized neurons involves the structural remodeling of dendritic branches including the occurrence, distribution, density, size, and shape of dendritic spines. Optimal degrees of synaptic connectivity (Litwin-Kumar et al., 2017) could be then associated with dendritic length and branching pattern, spine features, and the neuronal impedance, conductance, and voltage modulatory properties (Papoutsi et al., 2014). More specifically, spines are multifunctional integrative units (Shepherd, 1996) that increase the packing density of synapses by the convolution and interdigitation of cellular membrane, supporting more synapses without increasing the overall volume of the brain (Bourne and Harris, 2009). Dendritic spines provide an enhanced connectivity, modulation of synaptic processing, strength, and plasticity by considerably increasing the computational possibilities between cells (Anderson et al., 2009; Bourne and Harris, 2009; Rochefort and Konnerth, 2012; Yuste, 2013; Dall’Oglio et al., 2015; Tønnesen and Nägerl, 2016). Spines are specialized postsynaptic elements (see also Shepherd, 1996) that receive most (>90%) excitatory glutamatergic inputs (Rochefort and Konnerth, 2012; Yuste, 2013; Chen et al., 2014). Only a low percentage of spines (although particularly important, Müllner et al., 2015) is contacted by inhibitory γ-aminobutyric acid (GABA)-containing axon terminals (Kubota et al., 2007; Brusco et al., 2014).

Pyramidal neurons in neocortical layers III and V develop spines at different rates across the lifespan (Oga et al., 2017). In monkeys, the number of dendritic spines of pyramidal neurons in the primary visual cortex reduces following the onset of visual experience, whereas in areas of sensory association in the inferotemporal cortex and executive function in the granular prefrontal cortex grow more spines than they lose during the same period (Oga et al., 2017). Human pyramidal neurons also show an ontogenetic development that advances with remodeling dendrites and an increase in the spine number and complexity from the gestational period to birth and onward (Braak, 1980; Ramón y Cajal, 1909–1911; DeFelipe, 2011; Marín-Padilla, 2014). The density of dendritic spines in prefrontal pyramidal neurons have a developmental pruning and dynamic remodeling related to the reorganization of cortical circuitries during the first decades of the human lifespan (Petanjek et al., 2011) and a decline in spine measures latter (>50 years; Jacobs et al., 1997). That is, the human cerebral cortex shows neoteny and heterochrony in cortical circuits development and higher functions elaboration (Geschwind and Rakic, 2013). Furthermore, some pyramidal neurons depart from the general description that proximal dendritic segments are devoid of spines. Human pyramidal neurons can show dendritic spines distributed from proximal (e.g., 0–50 μm, Luengo-Sanchez et al., 2018) to long distal segments, as demonstrated below.

### Dendritic Spine Heterogeneity

According to morphological features, spines have been classified as stubby, wide, thin, mushroom-like, ramified, with a transitional aspect between these classes (as ‘protospines’ or ‘multispines,’ García-López et al., 2010), or “atypical” (also “multimorphic”) with a variety of different shapes, which includes double spines, spines with racemose appendages (with a lobed appearance and various bulbous enlargements and heads), and thorny excrescences (densely packed outgrowths showing fairly large spines with various round heads grouped around the stems) (Fiala and Harris, 1999; Arellano et al., 2007a; González-Burgos et al., 2012; González-Ramírez et al., 2014; Stewart et al., 2014; Dall’Oglio et al., 2015; Correa-Júnior et al., 2020). Small protrusions extending from the spine are spinules (Brusco et al., 2014; Vásquez et al., 2018), which are active zone-free invaginating structures that can participate in synaptic plasticity, including long-term potentiation (Petralia et al., 2018).

Spines are in a *continuum* of sizes and shapes and are found isolated or intermingled and forming groups (“clusters”) at the same dendritic branch, between different dendrites of the same neuron, or within the same subpopulation of neurons in a brain area (Fiala and Harris, 1999; Arellano et al., 2007a,b; Chen X. et al., 2011; Yuste, 2013; Rasia-Filho et al., 2012a; Rochefort and Konnerth, 2012; Brusco et al., 2014; Stewart et al., 2014; Dall’Oglio et al., 2015; Vásquez et al., 2018; Zancan et al., 2018). Adult human medial amygdaloid nucleus (MeA) neurons also show filopodium, large and thin dendritic spines with a gemmule appearance, and diverse synaptic arrangements as *en passant*, reciprocal, and serial ones (Dall’Oglio et al., 2015). At the ultrastructural level, spines can be monosynaptic or multisynaptic with contacts at the spine head and neck showing both asymmetric and symmetric characteristics (Dall’Oglio et al., 2015).

The relation between structure and function of the different dendritic spines for the fine-tuned synaptic processing is a matter of investigation and needs to be particularized for each sex, age, cell subpopulation, neural circuit, brain area, species, and specific natural or experimental circumstance (Benavides-Piccione et al., 2002; Arellano et al., 2007a; Bourne and Harris, 2008, 2009; Kasai et al., 2010; Rasia-Filho et al., 2012a; Rochefort and Konnerth, 2012; Yuste, 2013; Stewart et al., 2014; Dall’Oglio et al., 2015; Tønnesen and Nägerl, 2016; Lu and Zuo, 2017; Nakahata and Yasuda, 2018; Zancan et al., 2018). For example, some large dendritic spines can be more stable, have large postsynaptic density (PSD), and make strong connections. The size of the spine head scales with the size of the PSD, the presence and proportion of NMDA to AMPA glutamate receptors, and the amplitude of the excitatory postsynaptic current in mushroom-like spines with macular or perforated PSD (van der Zee, 2015 and references therein). In contrast, small spines would be transient forms (Woolfrey and Srivastava, 2016) and/or indicative of connections with a lower resistance to reach the parent dendrite (Segal, 2010). The length and width of the thin spine neck would determine the degree of electrical and biochemical compartmentalization of the spine (Noguchi et al., 2011; Yuste, 2013; reviewed in Tønnesen and Nägerl, 2016). Long necks in thin spines can impose more resistance and be plastic sites for synaptic coupling (Yuste, 2013).

Mushroom-like spines would standardize local postsynaptic potentials throughout the dendritic tree and reduce the location-dependent variability of excitatory responses (Gulledge et al., 2012). Other modeled distal synapses may not impact the cell’s output (Moldwin and Segev, 2019). Ramified spines have additional functional possibilities by displaying postsynaptic receptors on different parts of the spine heads (Verzi and Noris, 2009) with likely temporal and spatial specificity and signaling microdomains (Newpher and Ehlers, 2009; Chen and Sabatini, 2012). Synaptic amplification involving clustered dendritic spines would also enhance input cooperativity among coactive inputs at neighboring synapses (Harnett et al., 2012; Yadav et al., 2012), influencing network plasticity, learning, and memory (Frank et al., 2018; Kastellakis and Poirazi, 2019; for the dendritic mechanisms linking memories and overlapping allocations of synaptic resources see also Kastellakis et al., 2016).

These spine features add a high capacity of activity-dependent regulation and synaptic modulation for pyramidal neurons. This is corroborated by the (1) spatial distribution of spine types across proximal to distal branches; (2) extension and composition of the spine PSD; (3) differences in the composition and function of subcellular cytoskeleton, organelles (e.g., actin or smooth endoplasmic reticulum and mitochondria related to calcium levels modulation and initial synaptic establishment, respectively), dendritic mRNAs, and microRNA; and the (4) compartmentalization for both electrical (voltage coupling of spine and dendrite and vice-versa) and biochemical signals (e.g., affecting the diffusion rate of calcium, second messengers, and enzymes between dendritic shaft and spines) (Harris, 1999; Li et al., 2004; Rochefort and Konnerth, 2012; Yuste, 2013; Spruston et al., 2013; Stewart et al., 2014; Tønnesen and Nägerl, 2016; Hirsch et al., 2018). Because neighboring spines with varying shapes and sizes exist in the same dendritic shafts, “the morphological heterogeneity of spines, even in a small portion of the dendrite, is consistent with the idea that synaptic strength is regulated locally, at the level of each single spine” (Frick and Johnston, 2005; Arellano et al., 2007a,b; Chen X. et al., 2011; Lee et al., 2012; Dall’Oglio et al., 2015). Moreover, the presence of different spines in human pyramidal neurons “aligns well with emerging theoretical models of synaptic learning that demonstrate that synapses exhibiting a gradation of states, each bridged by distinct metaplastic transitions, bestow neural networks with enhanced information storage capacity” (Lee et al., 2012; Dall’Oglio et al., 2015 and references therein). These morphological features of human pyramidal neurons can reflect a more complex subcortical to cortical synaptic processing of sensory, emotional, and cognitive information adapted for species-specific social behaviors (Dall’Oglio et al., 2013, 2015). In summary, (1) most dendritic spines form synapses (Arellano et al., 2007b; see also Berry and Nedivi, 2017); (2) the presence and distribution of these spines are indicative of the neuronal connectivity (Cooke and Woolley, 2005; Chen X. et al., 2011; Chen et al., 2014); (3) spine number and shape implies various synaptic modulatory possibilities (Bourne and Harris, 2007; Yuste, 2013; Dall’Oglio et al., 2015; Tønnesen and Nägerl, 2016); (4) spines can have passive and active properties and their function affect the linear and non-linear neuronal processing of information (Oakley et al., 2001; Spruston et al., 2013; Brunel et al., 2014; Rollenhagen and Lübke, 2016); and (5) spines are cellular specializations with varied plasticity according to each brain area and species (Toni et al., 1999; Dall’Oglio et al., 2015; Hayashi-Takagi et al., 2015; Frank et al., 2018; Bucher et al., 2020).

### Implications for the Occurrence of Spiny Pyramidal Neurons

The development of cortical spiny pyramidal neurons has an evolutionary and ontogenetic value *per se* in terms of increased connectivity and integrated functions (Braak, 1980; Nieuwenhuys, 1994; Spruston, 2008; DeFelipe, 2011; Marín-Padilla, 2014; Sedmak et al., 2018; Petanjek et al., 2019). This provided a higher number of neuronal computational possibilities and increased the complexity of assembled cells in each specialized area even that they were limited by an anatomically restricted brain volume (Andersen et al., 2007; Geschwind and Rakic, 2013; Spruston et al., 2013; Marín-Padilla, 2014; Ramaswamy and Markram, 2015; Soltesz and Losonczy, 2018; Cembrowski and Spruston, 2019). Multiple spontaneous evolutionary changes would have increased numbers of neurons in the mammalian cerebral cortex and, although with differences toward primates, affected the average neuronal cell size, its dendritic and axonal arborization (Herculano-Houzel et al., 2014; Herculano-Houzel, 2019). In addition, “not only the increase in size” (i.e., number of cells), “of our brain seems to be responsible for our higher or more abstract mental abilities, but also the specialization of our cortical circuits appears to be critical” (DeFelipe, 2011).

With morphological and functional particularities, pyramidal cells are in the integrated subcortical to allocortical emergence of the limbic lobe, i.e., from allocortical areas with a primitive to three-layers organization advancing to the neocortical external and internal layers and subdivisions (detailed below). The arrangement of neurons into layers would represent a form of development of proper networks wiring length and space (Guy and Staiger, 2017; see also Chklovskii et al., 2002; Narayanan et al., 2017). However, the cortical functions rely on circuits specified by cell type composition and not only on a strict laminar classification (Guy and Staiger, 2017). In the human prefrontal cortex, pyramidal cell bodies have a cytoarchitectonic organization with stacks of 15–19 somata with apical dendrites arranged into vertically oriented bundles, as distinct clusters at the level of the layers III/V boundary, and forming minicolumns (Gabbott, 2003; see also Buxhoeveden and Casanova, 2002; Rockland, 2010). Pyramidal neurons vary in shape according to functional afferent and efferent features of different cortical loci (Anderson et al., 2009; Scholtens et al., 2014; Gilman et al., 2017; Cembrowski and Spruston, 2019), which change along with the human lifespan (Braak, 1980; Petanjek et al., 2008; Marín-Padilla, 2014; Sedmak et al., 2018; Soltesz and Losonczy, 2018). For example, the morphological complexity of layer V pyramidal neurons progressively increases from primary sensory to primary and supplementary sensory and motor cortices until association and multimodal ones (Kolb and Whishaw, 2015; Ramaswamy and Markram, 2015; see also Jacobs et al., 2001). This also relates to the cyto-, myelo-, receptor- and synaptic architecture of the neocortical layers, as well as differs between allocortical and isocortical areas (Palomero-Gallagher and Zilles, 2017).

## The Anatomical and Functional *continuum* for the Emergence and Development of Human Pyramidal Neurons

**FIGURE 1:**
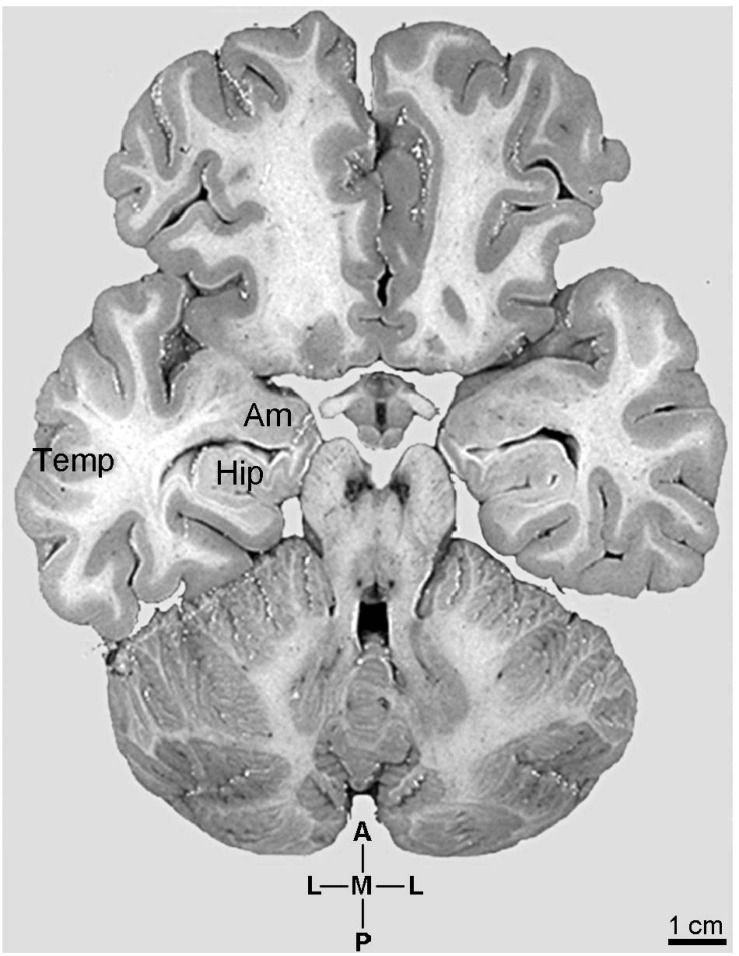
Photographic image of the human brain sectioned transversally at the level of the hypothalamic mammillary bodies to show the anatomical aspect of the continuum involving the emergence and development of pyramidal neurons in specific amygdaloid complex (**Am**) nuclei, hippocampus (**Hip**), and neocortical parietal lobe (**Temp**). Coordinates: A, anterior; L, lateral; M, medial; P, posterior. Adapted from the original image courtesy of the “Digital Anatomist Project.” Department of Biological Structure, Copyright, University of Washington, USA 1998 at http://da.si.washington.edu/cgi-bin/DA/imageform.

The anatomical *continuum* involving the human pyramidal neurons in the amygdaloid complex nuclei, in CA3 and CA1 hippocampal regions, and neocortex (parietal lobe) is exemplified in Figure 1. The morphological complexity of human pyramidal neurons varies from their emergence in the cortical (CoA) and basomedial (BM, but not in the MeA) nuclei of the amygdaloid complex toward the CA3 and CA1 hippocampal regions and the neocortical layers II–VI, with small pyramidal neurons in the upper layers II/III and large pyramidal neurons in the deep layer V. The following images were obtained with the Golgi-impregnation method adapted for the human *postmortem* brain (Dall’Oglio et al., 2010, 2013, 2015; Vásquez et al., 2018). Afterward, we proceeded to the 3D reconstruction of pyramidal neurons aiming further visualization and detailing of the dendritic spines from proximal to distal branches (Reberger et al., 2018; Vásquez et al., 2018; Correa-Júnior et al., 2020). Methodological advantages and technical constraints were outlined in previous reports (e.g., de Ruiter, 1983; Anderson et al., 2009; Dall’Oglio et al., 2010, 2013, Morales et al., 2014; Mohan et al., 2015; Tønnesen and Nägerl, 2016; Reberger et al., 2018).

Human pyramidal-like neurons are present in areas initially considered as subcortical ones (i.e., the amygdaloid complex). These cells can represent the place for the beginning of the limbic lobe (Heimer et al., 2008), phylogenetically and ontogenetically developed to provide further functional features for the amygdaloid and hippocampal areas toward the neocortical lobes and their subdivisions, including the anterior cingulate cortex (Wyss and van Groen, 1995; Gloor, 1997). In this context of “limbic areas” development, the cingulate cortex, part of the “proisocortex” in the paralimbic cortex, is phylogenetically older than the neocortex in the evolution of the mammalian brain (Braak, 1979; Kolb and Whishaw, 2015; Pandya et al., 2015). Nevertheless, by forming part of the neural circuitry for complex social processing, the cingulate cortex is much more than a primitive stage of cortical evolution (Allman et al., 2001; see further data in Vogt, 2015). It is a cytoarchitectonic and functional specialization of the neocortex with participation in emotion, interoceptive and visceral modulation, attention, cognition, and complex perceptions as self-awareness (Allman et al., 2001; Butti et al., 2013; Cauda et al., 2014; Correa-Júnior et al., 2020 and references therein). Likewise, the lateral parietal lobe adjacent to allocortical structures represents an evolved neocortical structure with primary, associative, and multimodal distributed functions (Nieuwenhuys et al., 1988; Miller and Vogt, 1995; DeFelipe, 2011; Pandya et al., 2015; Kolb and Whishaw, 2015).

Pyramidal-like neurons were found in the “amygdala.” However, the “amygdala” is neither an anatomical nor a functional unit (Swanson and Petrovich, 1998; see also Brodal, 1981; Heimer et al., 2008; LeDoux and Schiller, 2009). The amygdaloid complex represents a heterogeneous group of telencephalic nuclei and subnuclei studied according to cytoarchitectonic, neurochemical, connectional, and functional characteristics in different species (Johnston, 1923; Rasia-Filho et al., 2000; de Olmos, 2004; Schumann and Amaral, 2005; Heimer et al., 2008; Schumann et al., 2011; Dall’Oglio et al., 2013; Akhmadeev and Kalimullina, 2015; Janak and Tye, 2015; Olucha-Bordonau et al., 2015; Vásquez et al., 2018). The amygdaloid complex of mammals is composed of pallial (most nuclei and their subdivisions) and subpallial (the MeA and central “extended amygdala,” CeA) structures forming parallel circuits (Martínez-García et al., 2007). That is, the amygdaloid complex is composed of both cortical and subcortical origins (de Olmos, 2004; Medina and Abellán, 2012; Akhmadeev and Kalimullina, 2015; Olucha-Bordonau et al., 2015). Interestingly, pyramidal cells are found in brain areas that increased the processing of sensorial information from the environment and from conspecifics. The pyramidal neurons arising in such areas likely associated their cellular shape with more functional possibilities (and vice-versa). For the amygdaloid nuclei, relevant functions might have developed henceforth: (1) for perceiving and elaborating visual and auditory cues; (2) to attribute further emotional valence to these stimuli; (3) to modulate new memories and cognitive abilities; and (4) to expand the behavioral repertoire for complex social interactions between individuals, including judgments of facial expressions and emotional vocalization (Adolphs, 2003; Heimer et al., 2008; Rutishauser et al., 2015; Grisendi et al., 2019) likely contributing to parenting, empathy, happiness, fear, or disgust, for example (see relevant connectional and functional data in Diano et al., 2017). The advancement of both cellular and network processing capacities influenced the gain of species-specific features and adaptive responses. This improvement might lead the human brain networks to reach a higher level of magnitude and complexity from subcortical to cortical areas.

### The Amygdaloid Complex and “Cortical-Like Structures” Advancing to the Allocortex and Neocortex

**FIGURE 2:**
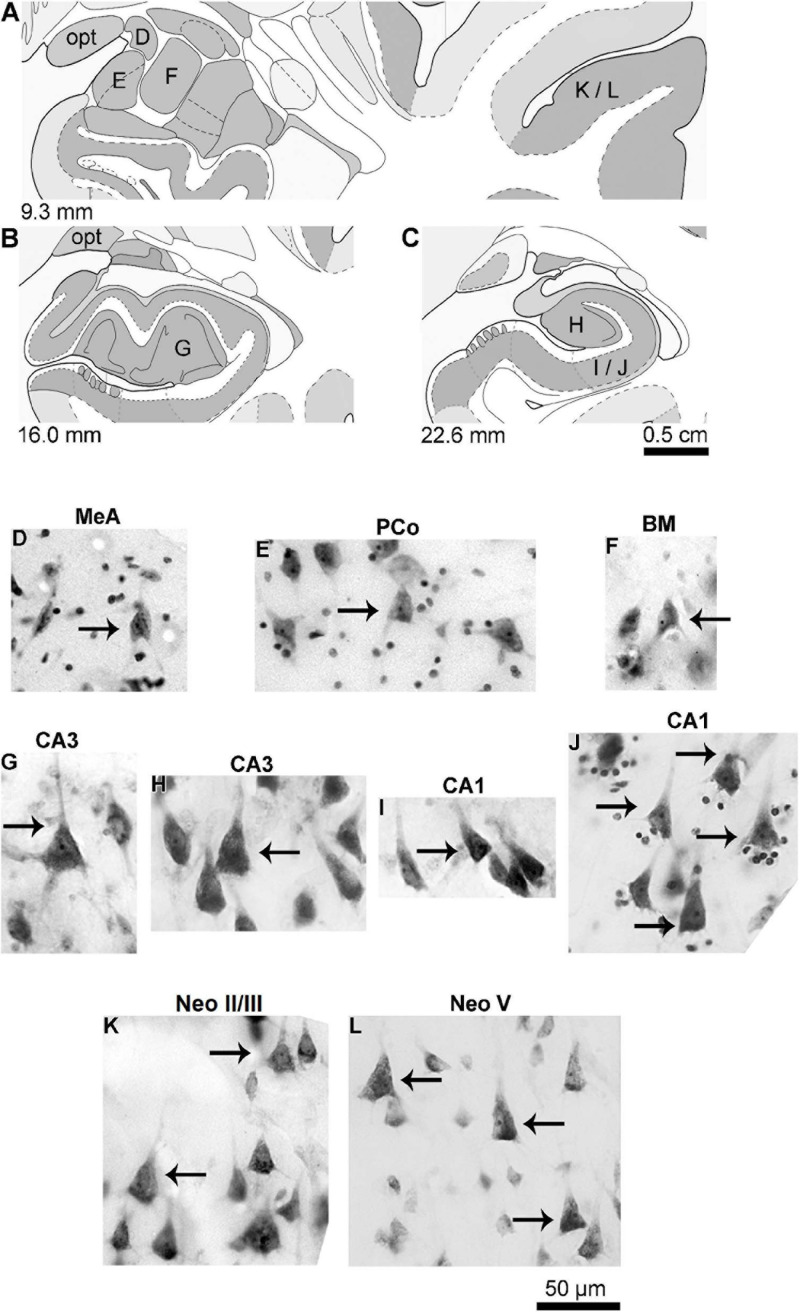
**(A–C)** Schematic diagrams of coronal sections (from 9.3 to 22.6 mm posterior to the midpoint of the anterior commissure; adapted from Mai et al., 2008) showing the location of the medial (MeA, **D**), cortical (posterior part, PCo, **E**), and basomedial (BM, **F**) amygdaloid nuclei, CA3 **(G,H)** and CA1 **(I,J)** hippocampal regions, and the temporal lobe neocortex layers II/III **(K)** and V **(L)** from where pyramidal neurons were studied in the human (adult male) brain. **(D–L)** Photomicrograph of thionine-stained cells in the studied brain areas. Arrows point to pyramidal neurons, except the angular neuron indicated in the medial amygdaloid nucleus **(D)**. Note the characteristic cell body shape of pyramidal neurons surrounded by other neuron types or small glial cells. Contrast and brightness adjustments were made with Photoshop CS3 software (Adobe Systems, United States). opt, optic tract.

The search for the emergence of pyramidal neurons led to the interface between nuclei of the amygdaloid complex and the hippocampal formation (Figures 1, 2A–C). Pyramidal neurons were also described in the subdivisions of the nucleus basalis of Meynert (based on Nissl staining and composing the cholinergic Ch4 cell group; Mesulam et al., 1983; Saper and Chelimsky, 1984; Liu et al., 2015). Three types of cerebral cortex have been studied: allocortex, periallocortex, and isocortex (Insausti et al., 2017). In rats, the pallial amygdala is considered an initial allocortical structure characterized by superficial layered cortical areas and deep non-layered parts (Olucha-Bordonau et al., 2015). This organization is recognized in the CoA by three cellular layers, i.e., an outer molecular layer (or layer 1), where terminates a direct projection from the olfactory bulbs, and two additional structurally different cellular layers (2 and 3; Olucha-Bordonau et al., 2015). Other nuclei show a cortical appearance and are associated with the olfactory tracts, as the bed nucleus of the accessory olfactory tract and the nucleus of the lateral olfactory tract (Olucha-Bordonau et al., 2015). These nuclei are interposed between the piriform and entorhinal cortices and the cortical amygdala as the rostral cortico-amygdala transition zone between the anterior cortical amygdala and the piriform cortex (Olucha-Bordonau et al., 2015). At the caudal edge of the amygdala, they compose the amygdalo-piriform transition area for the posterolateral cortical amygdala, the caudal piriform cortex, and the lateral entorhinal cortex (or, rather, an “amygdalo-entorhinal transition area”; Olucha-Bordonau et al., 2015).

Transition areas and/or specific nuclei with cellular components suggestive of a primitive cortex are found in the amygdaloid complex of primates (Amaral et al., 1992). In humans, Gloor (1997) included the prepiriform-periamygdaloid segments as part of the evolving mammalian allocortex, homologous with the ventral portion of the lateral pallium of amphibians and reptiles. The prepiriform cortex receives fibers from the lateral olfactory tract, and the periamygdaloid cortex hugs the “amygdala “(Gloor, 1997) medially to the “accessory basal nucleus” (i.e., the BM, Heimer et al., 2008; Janak and Tye, 2015; DiMarino et al., 2016) along the rostral two-thirds of its extent (Schumann and Amaral, 2005). In coronal sections of the human ventromedial area, at the level of the anterior amygdaloid region, it is possible to observe the close position of the prepiriform cortex dorsally, the rostral entorhinal cortex ventromedially, the cortico-amygdaloid transition area, and, at near caudal sections, first appearing, the lateral nucleus, BM, MeA, and CoA (Gloor, 1997). The phylogenetically ancient part of the amygdaloid complex includes both the MeA and CeA (Johnston, 1923). The MeA in the “extended amygdala” has a relatively smaller area than the other grouped nuclei in humans (de Olmos, 2004; Schumann and Amaral, 2005). The MeA occupies a superficial position and forms part of the medial border of the temporal lobe (with the CoA) in the part of the uncus represented by the gyrus semilunaris (Gloor, 1997). Both the MeA and CoA are regarded as “semicortex” in their superficial layers, and true subcortical structures in their deeper portions (Gloor, 1997 and references therein). The MeA subnuclei are involved with the (1) interpretation of environmental cues of conspecific stimuli; (2) processing of multiple sensory information, including direct and indirect olfactory and vomeronasal inputs with social relevance; (3) cellular responses to neural gonadal steroid actions for neuroendocrine secretion; and (4) modulation of reproductive and other social behaviors in rodents (Newman, 1999; Meredith and Westberry, 2004; Choi et al., 2005; Pro-Sistiaga et al., 2007; Rasia-Filho et al., 2012a,b; Petrulis et al., 2017; Petrulis, 2020). Being part of an organized neural network that projects to the bed nucleus of the stria terminalis (see relevant data on arousal behavior in Rodriguez-Romaguera et al., 2020) and to various hypothalamic and brainstem nuclei, the CeA and MeA subnuclei also participate in social and defensive reactions against innate and learned threats with neuroendocrine, behavioral, and sympathetic/parasympathetic responses to fearful and stressful stimuli (Davis, 1992; LeDoux, 1992; Quirk et al., 1995; Dayas et al., 1999; Rasia-Filho et al., 2000, 2012b; Petrovich et al., 2001; Marcuzzo et al., 2007; Neckel et al., 2012; Petrulis, 2020; Anilkumar et al., 2021). In humans, the extended amygdala responds to the emotional salience of positive and negative affect (Liberzon et al., 2003). The MeA projects to periallocortical, paleocortex, and archicortex, as well as to the insular agranular cortex and ventromedial prefrontal cortex (Everitt, 1995; de Olmos, 2004; Anderson et al., 2009; Petrulis, 2020). These data indicate that the MeA also participates, although with varied magnitude, in parallel circuits with different parts of the evolved neocortex (de Olmos, 2004) for social and emotional processing in our species (reviewed in Petrulis, 2020).

By contrast, the components of the basolateral nuclei are the largest amygdaloid group and, together with the CoA (Stephan et al., 1987), progressed most in size along with the mammalian evolution that led to primates (Gloor, 1997). These nuclei possess more than 50% of all neurons in the human amygdaloid complex (Schumann and Amaral, 2005). The basolateral group nuclei were considered “purely subcortical in location,” for none of them reach the surface of the uncus, although showing both cellular components of a “cortical-like” structure (Gloor, 1997) and development related to the allocortical piriform area (Johnston, 1923). The lateral and, afterward, the basal and the BM nuclei are the primary targets of cortical and subcortical afferent projections to the amygdaloid complex in primates (Kelly and Stefanacci, 2009; Janak and Tye, 2015). This developmental feature likely integrates the emergence of an anatomical and functional network from subcortical to allocortical connection endowed with further attributes for complex emotional, cognitive, and social behavior elaborations. For example, the lateral amygdala receives inputs from the hippocampal formation, thalamic and neocortical modality-specific sensory processing areas, integrate them, and display dynamic and plastic responses to signal danger as quickly as possible to initiate defensive behaviors without necessarily requiring additional neocortical processing (Quirk et al., 1995; Rasia-Filho et al., 2000 and references therein).

Neurons in the basolateral group of monkeys selectively change their firing rate by the perception of facial expressions and of specific parts of faces, such as the eyes (Rutishauser et al., 2015). In humans, a subset of amygdala neurons responds to information provided by individual parts of the eye and mouth region, the eyebrow, or wrinkles around the mouth, whereas another subset responds to the entire (whole) aspect of happy or fearful viewed faces (Rutishauser et al., 2011, 2013). This indicates that amygdaloid neurons receive and represent multi-modal sensory inputs for further biological significance and interpretation, relating them with the elaboration of the internal states and social interpretation evoked by faces, encoding the subjective judgment about the emotion perceived, and not only the objective features shown in the face (Wang et al., 2014; Rutishauser et al., 2015; Zheng et al., 2017). The attention associated with the response to stimulus novelty and the amygdalo-hippocampal communication during the encoding of emotional stimuli can be translated into memory and cognition (Rutishauser et al., 2015). The complexity of processing involving the human basolateral amygdala is further exemplified by its implication in late-life depressive symptom severity, associated with the dentate gyrus/hippocampal CA3 field and the lateral entorhinal cortex, during emotional episodic memory (Leal et al., 2017b).

Compared to rats, the large volume of the monkey amygdala (mainly due to the greater basolateral complex neuropil expansion than in the MeA and CeA) relates to a greater number of glial cells relative to neuron number, as well as more dendritic and axonal arborization in primates (Chareyron et al., 2011). Rats also have pyramidal neurons in specific amygdaloid nuclei, but these latter data indicate a higher capacity to process information by the primate amygdaloid cells and circuits. Furthermore, the dendritic arborization of pyramidal neurons in the CA1 hippocampal region is also higher in monkeys than in rats (Altemus et al., 2005). It is likely that the amygdaloid basolateral complex nuclei development parallels the cortical areas with which these nuclei are interconnected in primates (Chareyron et al., 2011), which includes reciprocal connections with the hippocampus (Janak and Tye, 2015), prefrontal cortex, anterior cingulate cortex (Freese and Amaral, 2009; Rutishauser et al., 2015), and primary sensory areas (Chareyron et al., 2011). That is, “the expansion of cortical areas and the greater complexity of cortical information reaching the amygdala are thus associated with a greater development of the amygdala nuclei interconnected with the neocortex” (Chareyron et al., 2011).

**FIGURE 3:**
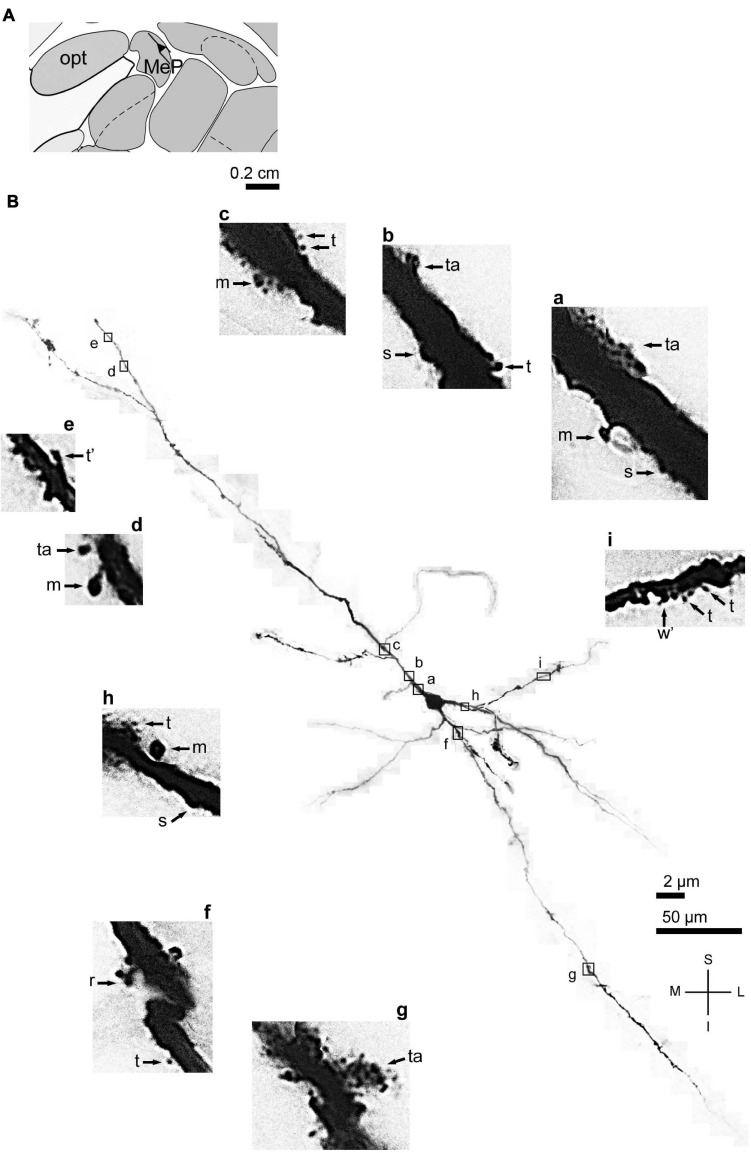
**(A)** Schematic diagram of a coronal section of the human brain showing the location where a Golgi-impregnated angular spiny neuron was observed (drawn in black) in the medial amygdaloid nucleus (posterior part, MeP; 9.3 mm posterior to the midpoint of the anterior commissure; adapted from Mai et al., 2008) opt, optic tract. **(B)** Digitized and reconstructed light microscopy image of a Golgi-impregnated angular neuron from the human (adult male) MeP. This multipolar cell is not a pyramidal neuron. Note the aspect of three primary dendrites and their length and ramification. The presence, distribution, and varied shapes of dendritic spines are shown in the corresponding inserts **(a-i)** at higher magnification. Dendritic spines were classified as stubby (s), wide (w), thin (t), mushroom (m), ramified (r), and atypical (a) with thorny excrescence aspect (in **g**). t* and w* = spinule in thin and wide spines, respectively. Contrast and brightness adjustments were made with Photoshop CS3 software (Adobe Systems, United States). I, inferior; L, lateral; M, medial; S, superior. Scales = 50 μm for the general view of the neuron and 2 μm for the inserts. Reprinted with permission (license # 4905550516803) from Dall’Oglio et al. (2015); Journal of Anatomy; Copyright 2015 John Wiley & Sons. Inc.

Accordingly, it was reasonable to hypothesize that human pyramidal neurons evolved in the amygdaloid basolateral complex and the CoA, but not in the MeA, while progressing onward to further allocortical and neocortical areas. One of the simplest histological approaches to visualize the presence of pyramidal neurons is the use of the Nissl/thionine staining (von Economo, 1927). Stained cells show a roughly triangular cell body shape, a spherical nucleus limiting the perikaryal cytoplasm, an evident nucleolus, and Nissl substance that can be present at the origin of primary dendrites, usually the apical one (Feldman, 1984; see Figures 2D–L). Former descriptions for the human MeA mention the presence of few “pyramidal-shaped cells” (Sims and Williams, 1990), “small neurons, some pyramidal, some fusiform or polygonal” (Gloor, 1997), and “pyramidal, multiangular, round, and spindle-shaped cells of different sizes” (de Olmos, 2004). de Olmos (2004) described “a tendency for the neurons of the medial nucleus to form layers, especially superficially, allowing the identification of a cell-poor molecular layer (L1), a superficial dense cell layer (L2), and a deep layer (L3) with somewhat less densely distributed neurons.” However, it was not possible to identify pyramidal neurons in the human MeA using the Golgi technique (Dall’Oglio et al., 2013, 2015). Local cells with a triangular cell body (Figure 2D) are not characterized by other relevant morphological features commonly associated with pyramidal cells, such as differences in the basal and apical dendritic thickness, length, and branching pattern (Dall’Oglio et al., 2013). Some of the primary dendrites in these spiny neurons resemble “main” processes extending in the neuropil, with long tapering shafts, and few branching points (Figure 3). These human MeA triangular neurons were named “angular” cells (Figure 3). They are one among other four Golgi-impregnated multipolar types in this nucleus (Dall’Oglio et al., 2013, 2015). Currently, it is not possible to affirm whether the human MeA neurons are “pure subcortical” or an evolutionary “older” form of (modified) pyramidal neurons that could be included as fusiform, “compass,” or multipolar cells according to the classification of Braak (1980). Golgi-impregnated neurons in the rat MeA subnuclei do not resemble pyramidal-like or classic pyramidal cells as well (Marcuzzo et al., 2007; reviewed in Rasia-Filho et al., 2012a,b). Two main types of multipolar neurons were described in the rat posterodorsal MeA: “bitufted” cells with two primary dendrites (i.e., they are not “bipolar” cells with a dendrite and an axon at opposite somatic sites) and stellate ones (with three or more primary dendrites). In addition, no evidence from electrophysiological data support typical pyramidal features for these two types of neurons in the posterodorsal MeA of rats (Dalpian et al., 2019), 50–90% of the total population of rat MeA neurons is GABAergic (Mugnaini and Oertel, 1985), and most of the efferent projections from the MeA are inhibitory GABAergic ones (Swanson and Petrovich, 1998).

**FIGURE 4:**
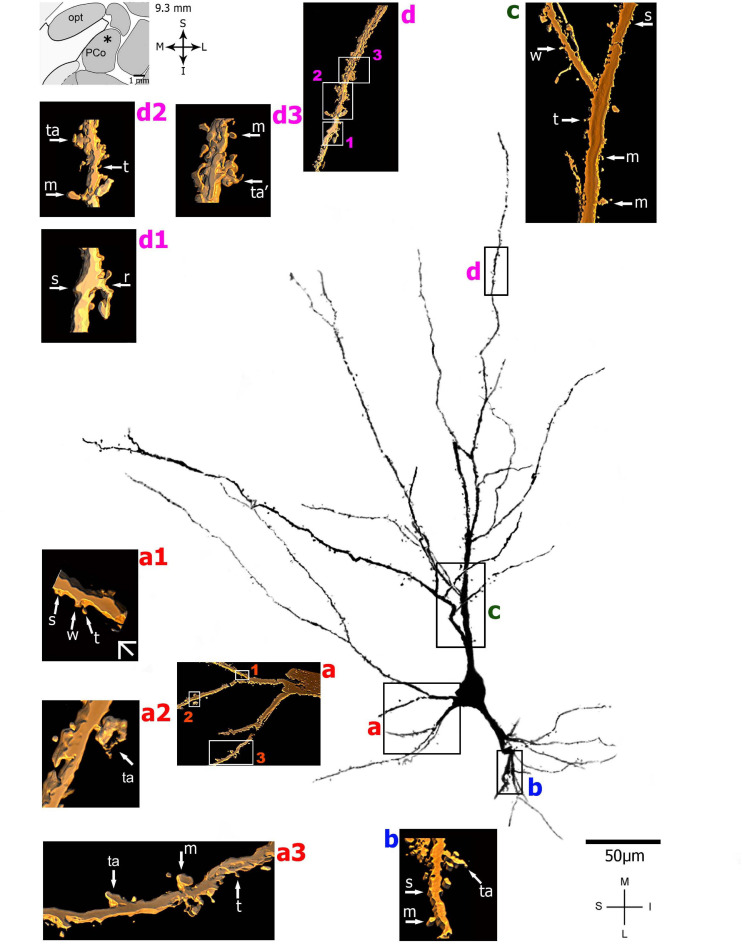
(Left top) Schematic diagram of a coronal section of the human brain showing the location where a Golgi-impregnated pyramidal-like neuron was observed (marked with an asterisk) in the cortical amygdaloid nucleus (posterior part, PCO, 9.3 mm posterior to the midpoint of the anterior commissure; adapted from Mai et al., 2008). opt, optic tract. (**Center and laterals**) Digitized and reconstructed light microscopy image of a Golgi-impregnated pyramidal-like neuron from the human (adult male) PCO. Note the aspect of two primary basal dendrites (marked **a**,**b**) and the primary apical dendrite **(c)**. The presence, distribution, and shape of 3D-reconstructed dendritic spines are shown in the inserts at higher magnification and correspond to the locations of **(a–d)**. Numbers accompanying these letters represent sampled segments of the respective dendrite (in **a1–a3** and **d1–d3**). There is a low density of pleomorphic spines in the proximal basal **(a)** and apical **(c)** dendrites and a moderate density in distal segments (**d1-d3**). Spines were classified as stubby (s), wide (w), thin (t), mushroom-like (m), ramified (r), or transitional/atypical ones (ta). The presence of a spinule is indicated graphically by the apostrophe attached to the corresponding spine (ta’ in **d3**). Contrast and brightness adjustments were made with Photoshop CS3 software (Adobe Systems, United States). I, inferior; L, lateral; M, medial; S, superior. Scale = 50 μm for the general view of the neuron and 2 μm for the inserts (the bar shown in **a1** applies to all other images of the 3D reconstructed dendritic branches and spines). This same procedure to demonstrate the 3D reconstructed dendrites and spines will be used for the next figures. Reprinted with permission (License Number 4554940100233) from Vásquez et al. (2018); The Journal of Comparative Neurology, Copyright 2018 Wiley Periodicals, Inc.

**FIGURE 5:**
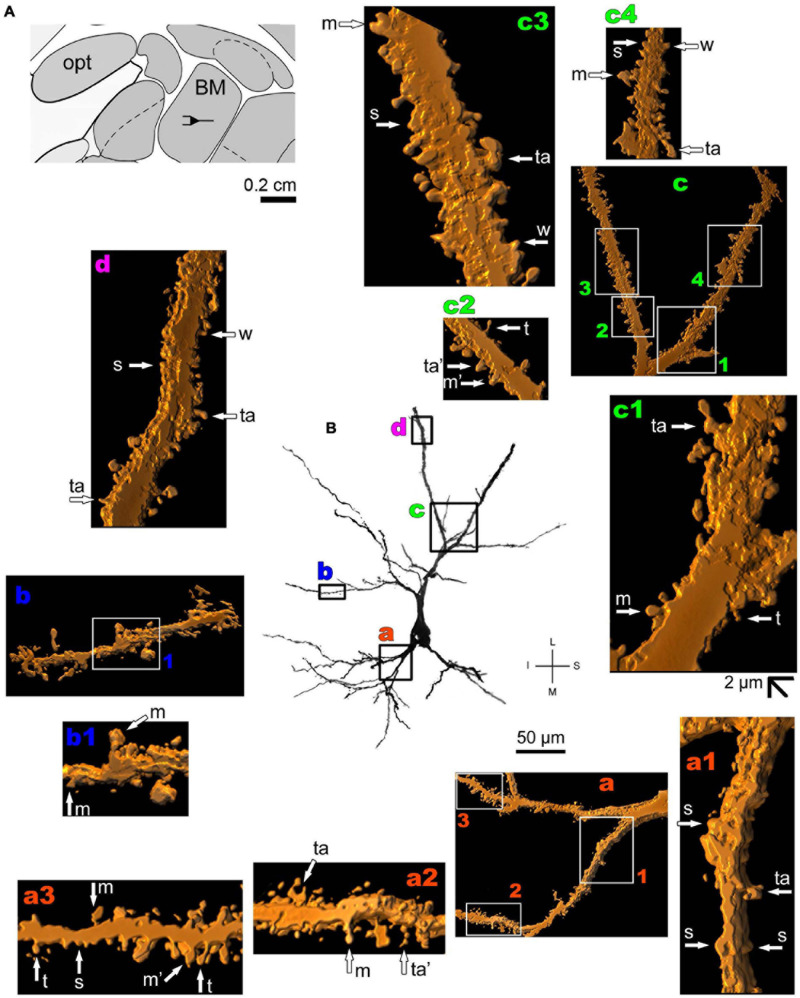
**(A)** Schematic diagram of a coronal section of the human brain showing the location where a Golgi-impregnated pyramidal-like neuron was observed (drawn in black) in the basomedial amygdaloid nucleus (BM, 9.3 mm posterior to the midpoint of the anterior commissure; adapted from Mai et al., 2008). opt, optic tract. (**B** and laterals) Digitized and reconstructed light microscopy image of a Golgi-impregnated pyramidal-like neuron from the human (adult male) BM. Note the aspect of the primary basal dendrites **(a)** and the primary apical dendrite oriented transversally in the section with its main ramification close to the cell body **(b–d)**. Compare this neuron with the other from the same BM region, but with a different apical dendrite branching aspect shown in Figure 6. The presence, distribution, and shape of 3D-reconstructed dendritic spines are shown in the inserts at higher magnification and correspond to the locations of **(a–d)**. Numbers accompanying these letters represent sampled segments of the respective dendrite (in **a1–a3,b1,c1–c4**). There is a high density of spines in the proximal basal dendrite **(a2,a3)** and along the intermediate to distal apical dendrites **(c1–c4,d)**. Spines were classified as stubby (s), wide (w), thin (t), mushroom-like (m) or transitional/atypical ones (ta). In a2, the m spine was identified after rotating the reconstructed image. The presence of a spinule is indicated graphically by the apostrophe attached to the corresponding spine (ta’ in **a2** and m’ in **a3**). Contrast and brightness adjustments were made with Photoshop CS3 software (Adobe Systems, United States). I, inferior; L, lateral; M, medial; S, superior. Scale = 50 μm for the general view of the neuron and 2 μm for the inserts (the bar shown in **c1** applies to all other images of the 3D reconstructed dendritic branches and spines).

**FIGURE 6:**
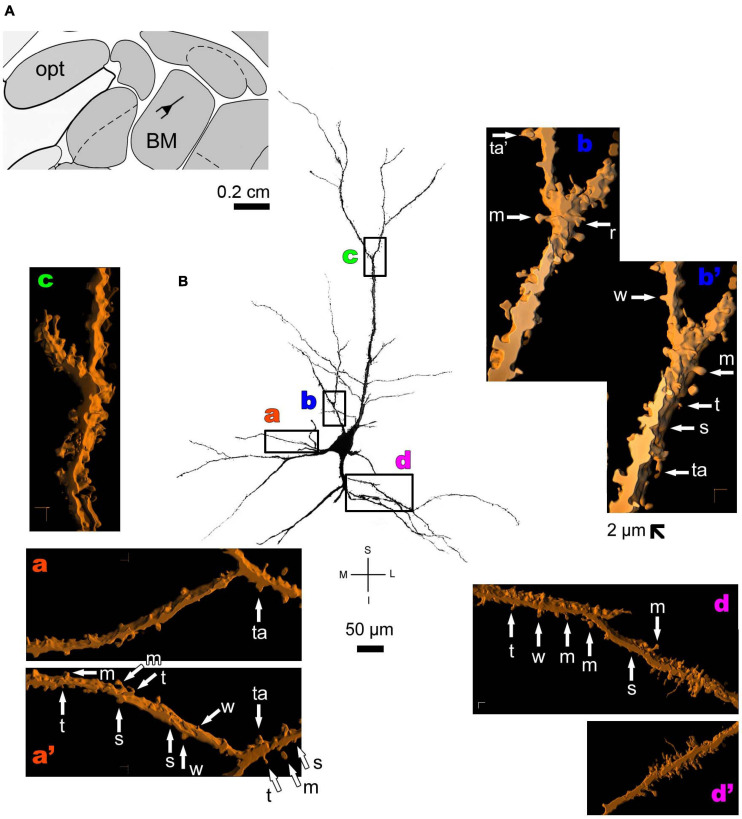
**(A)** Schematic diagram of a coronal section of the human brain showing the location where a Golgi-impregnated pyramidal neuron was observed (drawn in black) in the basomedial amygdaloid nucleus (BM, 9.3 mm posterior to the midpoint of the anterior commissure; adapted from Mai et al., 2008). opt, optic tract. (**B** and laterals) Digitized and reconstructed light microscopy image of a Golgi-impregnated pyramidal neuron from the human (adult male) BM. Note the aspect of the primary basal dendrites (**a,d**), proximal small branches **(b)**, and the primary apical dendrite oriented obliquely in the section showing its main ramification more distally from the cell body **(c)**. The presence, distribution, and shape of 3D-reconstructed dendritic spines are shown in the inserts at higher magnification and correspond to the locations of **(a–d)**. The apostrophe over these letters represent an image that was rotated in space after 3D reconstruction to detail the pleomorphic dendritic spines **(a’,b’,d’)**. There is a moderate **(a,a’)** to high **(d,d’)** density of spines in basal dendrites, proximal collaterals **(b,b’)**, and distal apical dendrites **(c)**. Spines were classified as stubby (s), wide (w), thin (t), mushroom-like (m), ramified (r) or transitional/atypical ones (ta). The presence of a spinule is indicated graphically by the apostrophe attached to the corresponding spine (ta’ in **b**). Contrast and brightness adjustments were made with Photoshop CS3 software (Adobe Systems, United States). I, inferior; L, lateral; M, medial; S, superior. Scale = 50 μm for the general view of the neuron and 2 μm for the inserts (the bar shown in **b’** applies to all other images of the 3D reconstructed dendritic branches and spines).

On the other hand, de Olmos (2004) described that “…many of the computations required to perform complex tasks are presumably initiated by the activation of neurons in the lateral nucleus” of the amygdaloid complex, which has pyramidal neurons (Sorvari et al., 1996). That is, both the human basolateral amygdaloid complex and the CoA have pyramidal-like and pyramidal neurons (Figures 2E,F, 4–6 and Supplementary Figures 1–3). Among nine other local neuronal types, Golgi-impregnated pyramidal-like cells in the human posterior CoA usually display a triangular soma, one main thick apical dendrite, and two basal dendrites of a similar thickness at their emerging points (Figure 4 and Supplementary Figure 1). The main apical shaft extends to the CoA surface and ramifies close to the cell body. Basal dendrites have variable branching points and lengths, some of them extending for a considerable distance away from the soma. There is an absence or low density of pleomorphic spines in the proximal dendritic shafts. The number of spines increases along the dendritic length to a moderate density. All types of dendritic spines (i.e., stubby, wide, thin, mushroom-like, ramified, or transitional/atypical ones) are observed, some with large and complex aspects and with a spinule (Figures 4a–d; Vásquez et al., 2018).

Thionine and Golgi staining data are congruent on the presence of pyramidal cells in the human BM (Braak and Braak, 1983). Both pyramidal-like (Figure 5 and Supplementary Figure 2) and pyramidal neurons (Figure 6 and Supplementary Figure 3) can be observed in this brain area. Pyramidal-like neurons show characteristic basal dendrites and a short apical dendrite branching close to the cell body (Figure 5), whereas pyramidal ones have a longer apical dendrite, various thin collateral branches, and a main ramification distally (Figure 6). In both cases, the apical dendrite may not be directed to the pial surface (Figures 5, 6). A high density of pleomorphic spines can be observed in the proximal basal dendrite and along the intermediate to distal apical dendrites in pyramidal-like neurons (Figures 5a–d). A moderate to a high density of spines in basal dendrites, proximal collaterals, and distal apical dendrites can occur in pyramidal neurons in the BM (Figures 6a–d).

**FIGURE 7:**
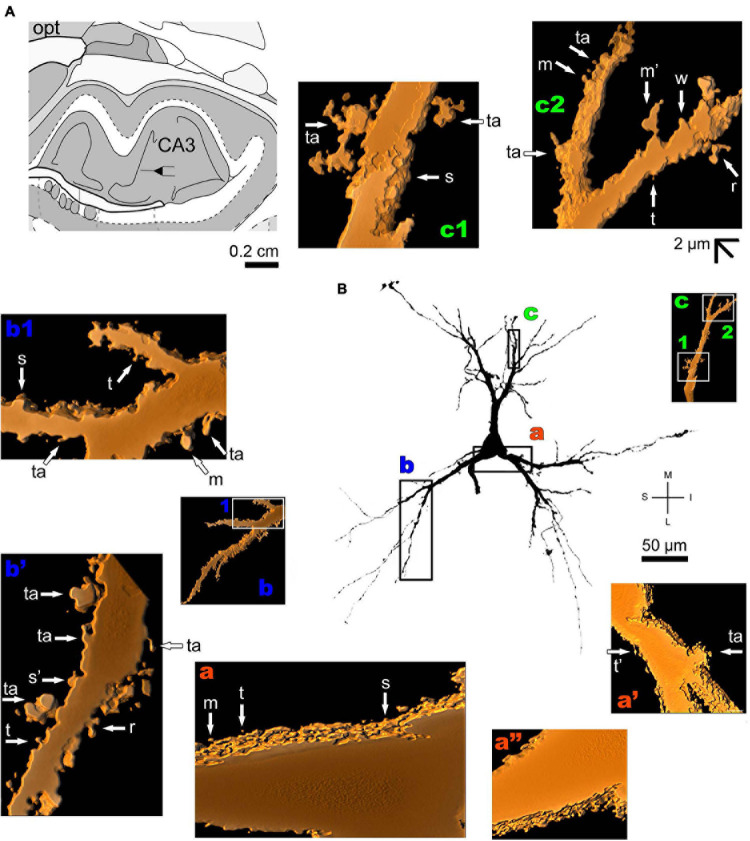
**(A)** Schematic diagram of a coronal section of the human brain showing the location where a Golgi-impregnated pyramidal neuron was observed (drawn in black) in the CA3 hippocampal region (16 mm posterior to the midpoint of the anterior commissure; adapted from Mai et al., 2008). opt, optic tract. (**B** and laterals) Digitized and reconstructed light microscopy image of a Golgi-impregnated pyramidal neuron from the human (adult male) CA3 hippocampal region. Note the aspect of the primary basal dendrites **(a,b)** and the short primary apical dendrite oriented to the medial surface of the brain with its main ramification close to the cell body **(c)**. The presence, distribution, and shape of 3D-reconstructed dendritic spines are shown in the inserts at higher magnification and correspond to the locations of **(a–c)**. Numbers accompanying these letters represent sampled segments of the respective dendrite (in **b1**,**c1,c2**). The apostrophe over the letters represents an image that was rotated in space after 3D reconstruction to detail the pleomorphic dendritic spines **(a’,a”,b’)**. There is a high density of small spines in the proximal basal dendrite **(a)** and a moderate to high density of pleomorphic spines in intermediate dendritic segments of basal **(b,b1,b’)** and apical dendrites **(c1,c2)**. Spines were classified as stubby (s), wide (w), thin (t), mushroom-like (m), ramified (r) or transitional/atypical ones (ta). Note the presence of thorny excrescences in both basal (ta in **a’**,**b’**) and apical (ta in **c1**) dendrites. The presence of a spinule is indicated graphically by the apostrophe attached to the corresponding spine (t’ in **a’,** s’ in **b’**, and m’ in **c2**). Contrast and brightness adjustments were made with Photoshop CS3 software (Adobe Systems, United States). I, inferior; L, lateral; M, medial; S, superior. Scale = 50 μm for the general view of the neuron and 2 μm for the inserts (the bar shown in **c2** applies to all other images of the 3D reconstructed dendritic branches and spines).

The human periallocortex (i.e., the presubiculum, parasubiculum, and entorhinal cortex) has pyramidal neurons (Insausti et al., 2017). The human CA3 hippocampal region contains pyramidal-like neurons or “short cortical pyramidal neurons” whose shapes are adapted to their position in the relatively small tissue volume. These cells can have primary thick basal dendrites and a short primary apical dendrite oriented to the medial surface of the brain. The main ramification of the apical dendrite is close to the cell body (Figure 7 and Supplementary Figure 4). There is a high density of small spines even in the proximal basal dendrites, and a moderate to high density of pleomorphic spines in intermediate dendritic segments of basal and apical branches, including the presence of thorny excrescences (Figures 7a–c; note this same kind of spine in the MeA angular neuron, Figure 3g; Dall’Oglio et al., 2015).

**FIGURE 8:**
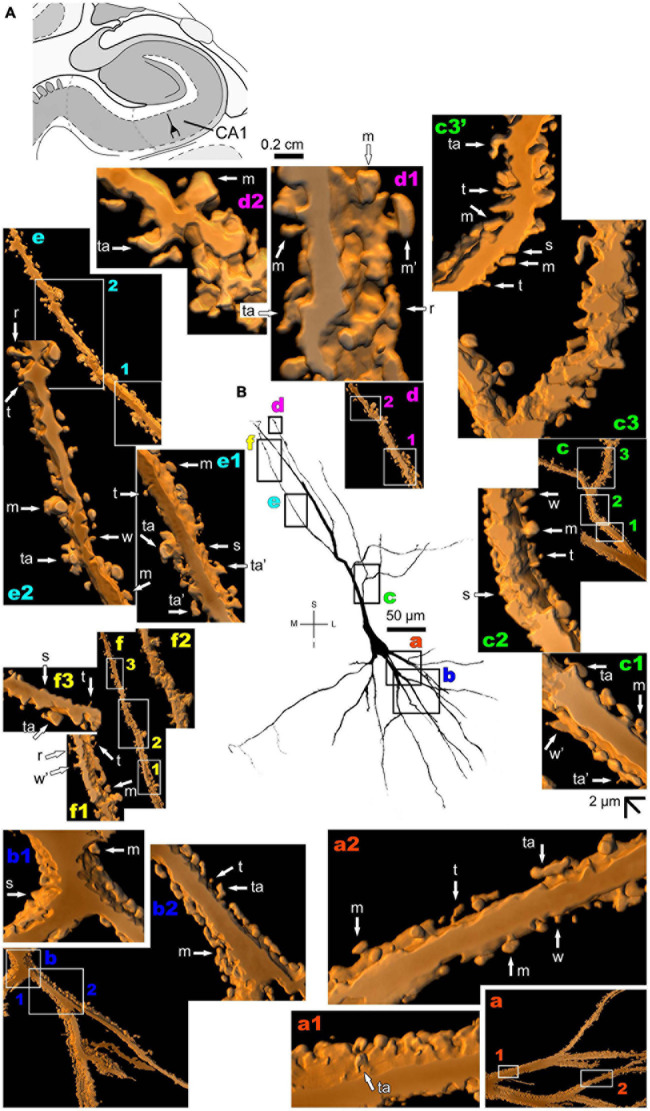
**(A)** Schematic diagram of a coronal section of the human brain showing the location where a Golgi-impregnated pyramidal neuron was observed (drawn in black) in the CA1 hippocampal region (22.6 posterior to the midpoint of the anterior commissure; adapted from Mai et al., 2008). opt, optic tract. (**B** and laterals) Digitized and reconstructed light microscopy image of a Golgi-impregnated pyramidal neuron from the human (adult male) CA1 hippocampal region. Note the aspect and length of the primary basal dendrites **(a,b)** and the main apical dendrite, oriented to the surface of the brain, tapering after collateral ramifications **(c–f)**. Compare this neuron with the next one from the same CA1 region with an apical dendrite that, after bifurcating, have long straight shafts with few collaterals as shown in Figure 9. The presence, distribution, and shape of 3D-reconstructed dendritic spines are shown in the inserts at higher magnification and correspond to the locations of **(a–f)**. Numbers accompanying these letters represent sampled segments of the respective dendrite (in **a1**,**a2**,**b1**,**b2**,**c1–c3,d1,d2,e1,e2,f1–f3**). The apostrophe over the letters represents an image that was rotated in space after 3D reconstruction to detail the pleomorphic dendritic spines **(c3’)**. There is an intermediate to high density of pleomorphic spines in the proximal segments of the basal **(a,b)** and apical **(c)** dendrites that continues toward distal segments in this latter **(d–f)**. Spines were classified as stubby (s), wide (w), thin (t), mushroom-like (m), ramified (r) or transitional/atypical ones (ta). Note the occurrence of different dendritic spines along the same segments, some relatively isolated (e.g., in **a2**,**e2**) and others in clusters (e.g., **b2**,**c3**,**d1**,**d2**). The presence of a spinule is indicated graphically by the apostrophe attached to the corresponding spine (w’ and ta’ in **c1,** s’ in **b1**, m’ in **d1**, ta’ in **e1**, and w’ in **f1**). Contrast and brightness adjustments were made with Photoshop CS3 software (Adobe Systems, United States). I, inferior; L, lateral; M, medial; S, superior. Scale = 50 μm for the general view of the neuron and 2 μm for the inserts (the bar shown in **(c1)** applies to all other images of the 3D reconstructed dendritic branches and spines).

**FIGURE 9:**
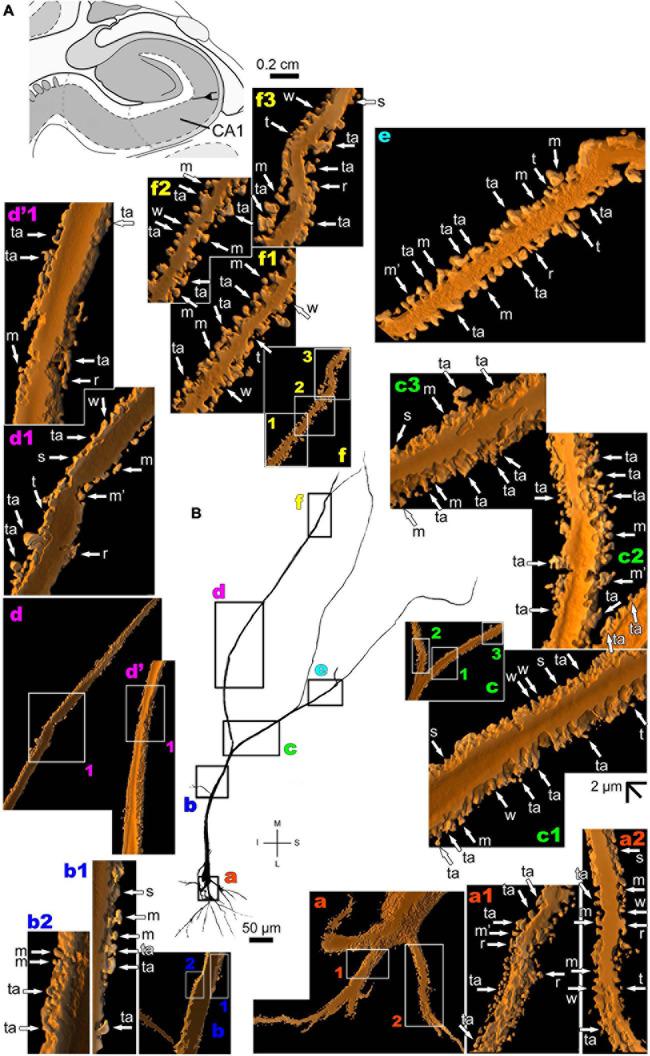
**(A)** Schematic diagram of a coronal section of the human brain showing the location where a Golgi-impregnated pyramidal neuron was observed (drawn in black) in the CA1 hippocampal region (22.6 posterior to the midpoint of the anterior commissure; adapted from Mai et al., 2008). opt, optic tract. (**B** and laterals) Digitized and reconstructed light microscopy image of a Golgi-impregnated large pyramidal neuron from the human (adult male) CA1 hippocampal region. Note the aspect and length of the primary basal dendrites **(a)** and the main apical dendrite oriented to the surface of the brain, which ramifies sparingly and have long straight shafts **(c–f)**. The presence, distribution, and shape of 3D-reconstructed dendritic spines are shown in the inserts at higher magnification and correspond to the locations of **(a–f)**. Numbers accompanying these letters represent sampled segments of the respective dendrite (in **a1**,**a2**,**b1**,**b2**,**c1–c3,d1,f1–f3**). The apostrophe over the letters represents an image that was rotated in space after 3D reconstruction to detail the pleomorphic dendritic spines **(d’,d’1)**. Note the high density of pleomorphic spines in the proximal segments of the basal dendrites **(a1,a2)**, the moderate to high density in spine density in the proximal segments of the apical dendrite **(b1,b2)**, the abundance of types and remarkably high density in intermediate apical segments **(c1–c3)**. Toward the distal parts of the apical dendritic branches, spines show an intermediate (**d1**,**d’1,e**) to an extremely high density of spines close to final shafts **(f1–f3)**. Dendritic spines were classified as stubby (s), wide (w), thin (t), mushroom-like (m), ramified (r) or transitional/atypical ones (ta). Dendritic spines of different shapes occur along the same segments (e.g., **a1**,**c1–c3**,**e**). The presence of a spinule is indicated graphically by the apostrophe attached to the corresponding spine (m’ in **a1,d1,e**). Contrast and brightness adjustments were made with Photoshop CS3 software (Adobe Systems, United States). I, inferior; L, lateral; M, medial; S, superior. Scale = 50 μm for the general view of the neuron and 2 μm for the inserts (the bar shown in **(c1)** applies to all other images of the 3D reconstructed dendritic branches and spines).

The human CA1 hippocampal region shows a variety of pyramidal shapes (Figures 8, 9 and Supplementary Figures 5, 6; see Benavides-Piccione et al., 2020 for additional morphological data). For example, some neurons can have basal and apical dendrites with a relative short aspect (Figures 8a–f and Supplementary Figure 5). Others, located at a deep position, have exceptionally long (at the order of millimeters), straight, and highly spiny apical dendrites with few ramifications (Figure 9 and Supplementary Figure 6). Basal dendrites may not be at opposite somatic poles in this kind of pyramidal neuron (Figure 9). In both short and long cells, the apical dendrite is oriented to the surface of the CA1 region. Basal and apical dendrites of local pyramidal neurons can be intermingled within the neuropil (Benavides-Piccione et al., 2020) and have pleomorphic spines (Figures 8a–f, 9a–f). The basal dendrites of the long pyramidal neuron show intermediate to a huge density of pleomorphic spines from proximal to distal segments (Figure 9a). The long apical branches also have a huge density of all types of spines, some with spinule (Figures 9b–f).

**FIGURE 10:**
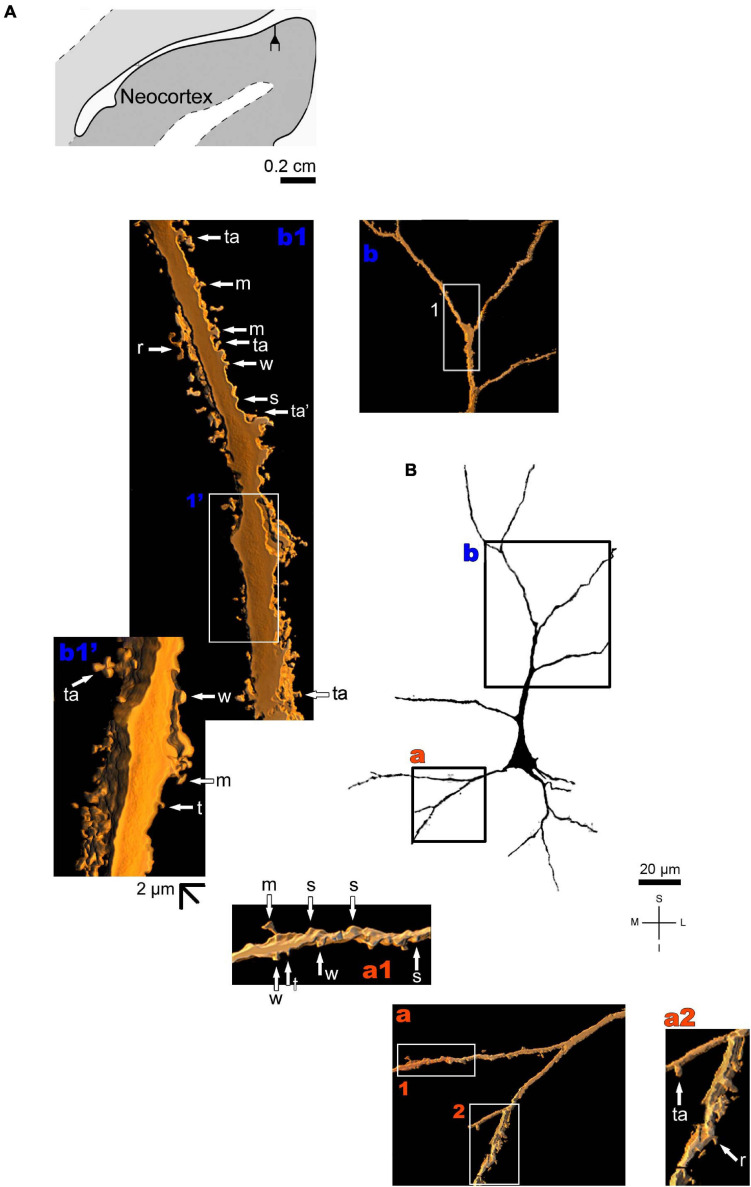
**(A)** Schematic diagram of a coronal section of the human brain showing the location where a Golgi-impregnated pyramidal neuron was observed (drawn in black) in the layer II/III of the anterolateral temporal lobe (Neocortex, 9.3 mm posterior to the midpoint of the anterior commissure; adapted from Mai et al., 2008). opt, optic tract. (**B** and laterals) Digitized and reconstructed light microscopy image of a Golgi-impregnated small pyramidal neuron from the external layers II/III of the human (adult male) anterolateral temporal neocortex. Compare the length and branching pattern of this neuron in the external pyramidal layer with the one in the internal pyramidal layer V shown in Figure 11. Note the aspect and branching pattern of the primary basal dendrites **(a)** and the apical dendrite oriented to the cortical surface **(b)**. The presence, distribution, and shape of 3D-reconstructed dendritic spines are shown in the inserts at higher magnification and correspond to the locations of **(a,b)**. Numbers accompanying these letters represent sampled segments of the respective dendrite (in **a1,a2**,**b1).** The apostrophe over these letters represent an image that was rotated in space after 3D reconstruction to detail the pleomorphic dendritic spines **(b1’)**. There is a low to moderate density of spines in basal dendrites **(a1,a2)** and a moderate to high density of spines in the apical dendrite **(b1,b1’)**. Spines were classified as stubby (s), wide (w), thin (t), mushroom-like (m), ramified (r) or transitional/atypical ones (ta). The presence of a spinule is indicated graphically by the apostrophe attached to the corresponding spine (ta’ in **b1**). Contrast and brightness adjustments were made with Photoshop CS3 software (Adobe Systems, United States). I, inferior; L, lateral; M, medial; S, superior. Scale = 20 μm for the general view of the neuron (compare to the other figures) and 2 μm for the inserts (the bar shown in **(b1’)** applies to all other images of the 3D reconstructed dendritic branches and spines).

**FIGURE 11:**
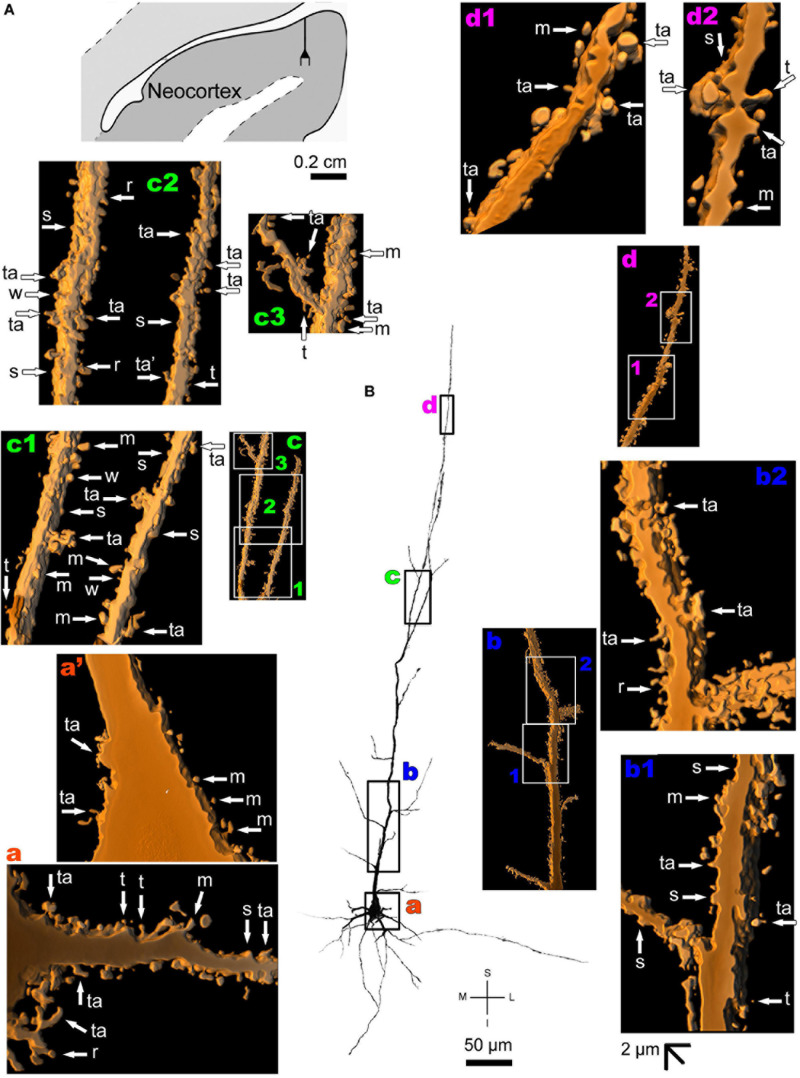
**(A)** Schematic diagramof a coronal section of the human brain showing the location where a Golgi-impregnated pyramidal neuron was observed (drawn in black) in the layer V of the anterolateral temporal lobe (Neocortex, 9.3 mm posterior to the midpoint of the anterior commissure; adapted from Mai et al., 2008). opt, optic tract. (**B** and laterals) Digitized and reconstructed light microscopy image of a Golgi-impregnated large pyramidal neuron from the internal layer V of the human (adult male) anterolateral temporal neocortex. Note the aspect and length of the primary basal dendrites **(a)** and the main apical dendrite oriented to the surface of the brain with collateral branches and a long straight vertical shaft **(b–d)**. The presence, distribution, and shape of 3D-reconstructed dendritic spines are shown in the inserts at higher magnification and correspond to the locations of **(a–d)**. Numbers accompanying these letters represent sampled segments of the respective dendrite (**b1**,**b2**,**c1–c3,d1**,**d2**). The apostrophe over the letters represent an image that was rotated in space after 3D reconstruction to detail the pleomorphic dendritic spines **(a’)**. Note the moderate density of pleomorphic spines in the proximal segments of the basal dendrites **(a,a’)** and apical dendrite **(b1)**. Moderate to high density of dendritic spines are observed toward intermediate (**b2**) to distal **(c1–c3)** segments of the apical dendrite. Moderate spine density is observed in more distal dendritic shaft **(d1,d2)**. Some spines were observed in the cell body **(a’)**. Dendritic spines were classified as stubby (s), wide (w), thin (t), mushroom-like (m), ramified (r) or transitional/atypical ones (ta). As in the other pyramidal neurons, dendritic spines of different shapes and sizes occur along the dendritic segments (e.g., **a**,**b2**,**c1**,**d1**). Contrast and brightness adjustments were made with Photoshop CS3 software (Adobe Systems, United States). I, inferior; L, lateral; M, medial; S, superior. Scale = 50 μm for the general view of the neuron and 2 μm for the inserts (the bar shown in **(b1)** applies to all other images of the 3D reconstructed dendritic branches and spines).

The human neocortex sampled (i.e., the anterolateral temporal lobe) displays a short, small pyramidal neuron in the superficial layers II/III (Figure 10 and Supplementary Figure 7). Basal dendrites branch sparingly and show a low to moderate density of pleomorphic spines (Figure 10a). The short apical dendrite has few collaterals, is oriented to the cortical surface, and displays a moderate to high density of spines (Figure 10b). Pyramidal neurons in the deep layer V have basal dendrites ramifying horizontally or directed to the adjacent layer VI. The apical dendrite is a long and straight main vertical shaft oriented to the superficial layers with some collateral branches (Figure 11 and Supplementary Figure 8). There is a moderate density of pleomorphic spines in the proximal segments of the basal dendrites (Figure 11a). Spines in the apical dendrite show a moderate to high density from proximal to intermediate segments (Figures 11b,c) and a moderate density distally (Figure 11d).

## Vulnerability of Human Pyramidal Neurons

Although not reductionist, the development of further neural abilities and conscious emotional processing by neural circuits enabled emergent properties with integrated neurophysiological, physicochemical, and mathematical/statistical possibilities. Our evolved nervous tissue organization provided complex motor and social abilities (for speaking, manipulating fire, agricultural techniques, domestication of animals, etc.); language and knowledge transmission between generations (mathematics, medicine, navigation, etc.); abstract thinking, creativity, and artistic expressions (philosophy, painting, creating and playing musical instruments, etc.); technology advancement and various other implications for the human behavior (e.g., see Bunge, 1980; Searle, 1997; Elston, 2003; Persinger and Koren, 2007; DeFelipe, 2011; Geschwind and Rakic, 2013; Marín-Padilla, 2014; Aru et al., 2019; Herculano-Houzel, 2019). The specialized neurons and circuits that provided these higher abilities also show vulnerabilities and are related to complex disorders with variable clinical manifestations in humans (see a parallel discussion and examples in Clowry et al., 2010; Butti et al., 2013; Geschwind and Rakic, 2013; Cauda et al., 2014; Hodge et al., 2019; Correa-Júnior et al., 2020). This implicates the amygdalo-neocortical *continuum* in a broad range of neurological and psychiatric conditions affecting memory, cognition, and mood dysfunction (Carlo et al., 2010; Schumann et al., 2011), as well as in language and social awareness disorders (Yudofsky and Hales, 2004; Geschwind and Rakic, 2013; also see Herculano-Houzel, 2019).

As highlighted by Heimer et al. (2008) for the Vogt’s theory of “Pathoklise und Pathoarchitektonik,” “certain physiochemical properties of nerve cells that share common morphological characteristics, and often constitute cytoarchitectonically definable areas, confer upon them specific susceptibilities to a variety of pathogenic agents” (Vogt and Vogt, 1922; Gloor, 1997). For example, cortical parts of the greater limbic lobe are “disproportionally targeted by neurofibrillary tangles in Alzheimer’s disease, and there is good evidence that this degenerative disease begins in selective non-isocortical parts” within it. “We favor the inclusion of the laterobasal-cortical complex of the amygdala in the limbic lobe. Parts of it are cortex, and the various laterobasal nuclei contain cortical-like neurons aggregated into nuclei segregated by the intrinsic white matter of the amygdala. Moreover, chemoarchitectonic characteristics and connectional patterns. support this viewpoint. The inclusion of the laterobasal amygdaloid complex in the limbic lobe is supported by developmental investigations…, which indicate that the laterobasal-cortical amygdala develops in association with the nearby populations of neuronal precursors that ultimately form the cortical mantle. By exclusion, the remaining amygdala, the centromedial complex, belongs to the extended amygdala” (Heimer et al., 2008 and references therein). The current morphological data on the occurrence of pyramidal-like and pyramidal neurons shown here are in line with this former proposition.

### Alzheimer’s Disease

The amygdaloid nuclei (as well as the allocortical and neocortical areas) that are sites of the emergence and development of pyramidal neurons are also coincident with the neuropathological findings of AD (Morrison et al., 1987; Heun et al., 1997; Merino-Serrais et al., 2011). This is the case for the particularly vulnerable and most severely affected lateral amygdaloid nucleus (de Olmos, 2004), CoA, periamygdaloid cortex (Schmidt et al., 1996), specific pyramidal layers of the parahippocampal region and hippocampus, and functionally organized hierarchical areas of the neocortex (Morrison et al., 1987). Subpopulations of pyramidal neurons with specific anatomical and molecular profiles may show a differential vulnerability in AD (Morrison et al., 1987; Hof and Morrison, 1990; Hof et al., 1990). These neurons display in common high intracellular levels of non-phosphorylated neurofilament protein and a long axonal projection that terminates within the neocortex, hippocampus, or related telencephalic structures (including cells from the locus coeruleus and the nucleus basalis of Meynert; Morrison et al., 1987).

In the context of cellular vulnerability (Morrison et al., 1987; Hof et al., 1990; Braak and Braak, 1991), pyramidal neurons show atrophy of basal and apical dendrites and reduction in the number of dendritic spines with the AD progression (Penzes et al., 2011; Overk and Masliah, 2014). There is a notable atrophy of the amygdaloid nuclei and hippocampus, but also in the nucleus accumbens, putamen, and thalamus (Benzinger et al., 2013), an issue open to debate on secondary atrophy due to reduced connectivity. Neurodegeneration advancing in the limbic lobe harms the dendrites and spines of pyramidal neurons in the subiculum, the CA1 hippocampal region, and the entorhinal cortex in the mesial temporal lobe, further progressing to involve the nucleus basalis of Meynert and associative areas in the frontal, parietal, and temporal lobes (Hyman et al., 1984; Saper and Chelimsky, 1984; Braak and Braak, 1991; Ishunina and Swaab, 2001; Thompson et al., 2001; Peçanha and Neri, 2007; Serrano-Pozo et al., 2011; Liu et al., 2015). Pyramidal neurons in layers III to VI are severely affected in different parts of the neocortex (Hof and Morrison, 1990; Hof et al., 1990; Arnold et al., 1991; Braak and Braak, 1991). There is also a selective loss of the giant cells of Meynert in the superficial part of layer VI in the human visual cortex (Hof and Morrison, 1990). On the other hand, the corticospinal-projecting Betz cells in motor cortex are not involved in the AD pathology (Morrison et al., 1987).

Brain tissue atrophy along with synaptic dysfunction or loss is linked with symptomatic memory and cognitive decline in AD (Heun et al., 1997; Yudofsky and Hales, 2004; Serrano-Pozo et al., 2011; Dorostkar et al., 2015). However, current experimental, imaging, and biomarkers data provided other important pieces to this scenario of progressive cortical damage. Although not all regions have hypometabolism and atrophy at the same time (Benzinger et al., 2013), it is important to consider that cortical brain circuits can be morphologically and functionally impaired even in the early stages of AD (Huijbers et al., 2015; Palmqvist et al., 2017; Leal et al., 2018). The β-amyloid peptide (Aβ)-related neuroinflammation involves microglial dysfunction and a feedforward harmful cycle in mice (Friker et al., 2020). The accumulation of Aβ begins slowly, years before biomarkers become abnormal (Leal et al., 2018). Nearly every cortical region, with relative initial sparing of entorhinal, precentral, and postcentral cortices, show Aβ deposition 15 years before the expected onset of the symptomatic phase in individuals with autosomal dominant AD (Benzinger et al., 2013). Reduced cortical glucose metabolism and cortical thinning occur 10 and 5 years before the onset of symptoms in the precuneus/posterior cingulate/lateral parietal cortex and in the middle temporal gyrus/lateral prefrontal cortex, respectively (Benzinger et al., 2013). Afterward, atrophy becomes evident in the precuneus, entorhinal, lateral temporal, and lateral parietal cortices, whereas the anterior cingulate cortex increases its thickness (Benzinger et al., 2013).

It is worth noting that Aβ fibrils start to accumulate preferentially in the precuneus, medial orbitofrontal cortex, and posterior cingulate cortex of subjects at preclinical AD stage (Palmqvist et al., 2017). About half of the patients with a mild cognitive impairment show high levels of cortical Aβ deposition, which is more than the percentage of Aβ^+^ clinically normal older adults (Huijbers et al., 2015). Aβ accumulating APP/PS1 transgenic mice show altered dendritic spines in the hippocampal CA1 *stratum oriens* and *stratum radiatum* with layer-specific decrease in spine neck length and increase of spines with a small head volume (Merino-Serrais et al., 2011). In humans, the initial Aβ accumulation can harm synaptic transmission and is associated with hypoconnectivity between the areas of the “default mode network” and the frontoparietal network (Palmqvist et al., 2017). Autosomal dominant AD mutation carriers also show an inverse correlation between regional and global amyloid deposition and cerebral blood flow as they approached the age of dementia diagnosis (Yan et al., 2018). Amyloid may exacerbate cognitive and vascular dysfunction by a tau-mediated pathway (Albrecht et al., 2020). Aβ^+^ patients continue toward AD dementia showing high levels of hippocampal activity, increased rates of hippocampal atrophy, and progressive decline in global cognition (Huijbers et al., 2015). That is, the increased hippocampal activity is associated with the levels of Aβ deposition, follows an initial aberrant activity (decreased deactivation) in the default network, and might reflect compensatory neuronal activity striving against memory impairment and/or local excitotoxicity caused by the accumulation of soluble Aβ (Huijbers et al., 2015). The greater hippocampal activation can be detrimental, associated with further Aβ deposition, and cognitive decline (Leal et al., 2017a). Patients with AD can also have subclinical epileptiform activity, indicative of sustained neuronal and network hyperexcitability, while they are at risk for accelerated deleterious effects on cognition (Vossel et al., 2016). Thus, the progression of AD demands the earliest possible detection and treatment of pathological changes before cognitive impairments begin to occur in vulnerable cortical areas (Leal et al., 2018; see additionally Leng et al., 2021).

### Temporal Lobe Epilepsy

Temporal lobe epilepsy (TLE) is one of the most common form of focal epilepsy refractory to antiepileptic drugs (Semah et al., 1998; Wiebe, 2000; Kumlien et al., 2002; Téllez-Zenteno and Hernández-Ronquillo, 2012). Seizure-generation and propagation involve the network architecture of limbic structures and neocortical brain regions for the hypersynchrony and hyperexcitability activity, with the midline thalamus serving as a synchronizer (Bertram et al., 1998; Bertram, 2009; Vismer et al., 2015; Wicker and Forcelli, 2016; see additional brain areas in Arakaki et al., 2016; Soper et al., 2016; Wicker et al., 2019). The threshold for kindling to induce limbic seizures in animal models of TLE is significantly lower in structures with pyramidal neurons, such as the amygdaloid complex, hippocampus, entorhinal cortex, piriform cortex (and endopiriform nucleus), olfactory cortex, and interconnected neocortical parts (Gloor, 1997; Vismer et al., 2015; Insausti et al., 2017) when compared to the kindling of other areas, such as the thalamic midline nuclei. Local and remote assemblage of neurons and their microcircuits play an important role in seizure initiation and spreading (Du et al., 1993; Hudson et al., 1993; Natsume et al., 2003; Bertram, 2009; Vismer et al., 2015; Wicker and Forcelli, 2016). For example, the ventrorostral aspect of the piriform cortex encompasses a chemoconvulsant trigger zone particularly named as “*area tempestas*” (reviewed in Vismer et al., 2015).

The amygdaloid complex, the hippocampal formation, and other associated limbic structures are important sites for seizures generation in TLE (Bertram et al., 1998; Mangan et al., 2000; Vismer et al., 2015; Wicker and Forcelli, 2016). In some of them, seizure onset is easier to detect than in others (Bertram et al., 1998; Bertram, 2009). In the hippocampus, which is a laminated cortical structure, the orientation of the pyramidal cell layer generates powerful electrical fields that can be detected by volume conduction at some distance. In addition, the pyramidal neurons in the hippocampus are the main mediators of excitatory transmission (Andersen et al., 2007). They receive excitatory inputs and, in turn, make equally powerful excitatory glutamatergic synapses with their target neurons in and outside of the hippocampal formation (Gloor, 1997). The amygdaloid complex is an important site of seizure onset in TLE (Gloor, 1997). Although some amygdaloid nuclei have pyramidal neurons and are another important site of seizure onset in TLE (Gloor, 1997), these amygdaloid cells appear to be not organized in polarized layers as the hippocampus or neocortex, and the electrical field created by these neurons’ discharges generate just a small volume conduction outside the structure itself. Nevertheless, specific involvement of amygdala on seizure onset in humans was first observed by Feindel and Penfield (1954) during stimulation of periamygdaloid region in an awake, locally anesthetized patient during a surgery to treat refractory epilepsy. Basolateral amygdala hyperexcitability has also been demonstrated in the chronic kainic acid model in rats (Smith and Dudek, 1997). *In vitro* studies showed that multiple limbic sites (including the basolateral amygdala) have epileptiform discharges associated with prolonged depolarizations and multiple superimposed action potentials (Bertram et al., 1998; Fountain et al., 1998; Bertram, 2009). It remains to be settled whether pyramidal neurons in the human amygdaloid complex have the same electrophysiological properties as other allocortical and neocortical regions and/or behave correspondingly during TLE.

Additional studies with experimental models of TLE and in patients with refractory TLE demonstrated the important participation of the hippocampus on both seizure onset (Yaari and Beck, 2009 and references therein) and maintenance of seizure activity (Alonso-Nanclares et al., 2011) with pronounced changes in intrinsic properties of CA1 pyramidal cells (Jensen and Yaari, 1997; Sanabria et al., 2001; Beck and Yaari, 2008; Chen S. et al., 2011). In hippocampal slices from patients with refractory TLE, the activity of subicular pyramidal cells is associated with epileptiform activity generation (Wozny et al., 2005; Wittner et al., 2009). Most CA2 pyramidal cells can fire spontaneously, depolarize during interictal-like events, and generate independent epileptiform activity (Wittner et al., 2009). Altered expression or modulation of ion channels, can result in abnormal membrane depolarization, such as an up-regulation of T-type Ca^++^ current in dendrites and down-regulation of dendritic I_A_ that affect the magnitude of the corresponding currents and change the neuronal firing pattern from regular to burst mode (Yaari et al., 2007; Remy et al., 2010). This firing behavior like in the neocortical intrinsically bursting pyramidal cells, under the effect of GABAergic antagonists and threshold stimulation of afferent fibers, can evoke long-latency epileptiform bursts (Tasker et al., 1992). Changes in dendritic ion channels of CA1 pyramidal cells also affect the dynamic of excitatory postsynaptic responses (EPSPs) generated at dendritic sites and the backpropagation of action potentials into the dendritic tree. These dendritic ion channels can be activated by subthreshold EPSPs for spike initiation (Yaari and Beck, 2009 and references therein). The number of axon collaterals of CA1 pyramidal cells also increases in pilocarpine treated rats and in patients with TLE, which indicates that a network reorganization in CA1 contributes to local hyperexcitability via increased backward excitation (Lehmann et al., 2000). The burst discharges of intrinsically bursting CA1 pyramidal neurons can recruit additional neurons via recurrent excitatory connections, contributing to the generation of epileptic discharges (reviewed in Yaari and Beck, 2009). Aberrant synaptic reorganization is evident in the glutamatergic, zinc-containing mossy fibers of granular cell of dentate gyrus and CA3 pyramidal cells as well (Sutula et al., 1988).

Besides the recurrent excitation, selective degeneration of highly vulnerable hippocampal CA1 and CA3 pyramidal neurons is a common structural change related to epilepsy and other neurological disorder as AD and stroke (Medvedeva et al., 2017). Several lines of evidence suggest that glutamate is the neurotransmitter involved in this hippocampal neurodegeneration (reviewed in Lewerenz and Maher, 2015). High levels of glutamate are toxic to select groups of pyramidal cells and, when in subtoxic levels, reduce pyramidal neuron dendrites (Mattson et al., 1989). All zinc-containing neurons are glutamatergic but not all glutamatergic neurons contain zinc (Frederickson et al., 1990). Several glutamatergic releasing pyramidal cells can corelease zinc as those in the CA3 and CA1 regions, prosubicular, piriform cortex, and neocortical layers II-IV and VI (Frederickson et al., 2000; Takeda et al., 2006). With a few exceptions, zinc-containing neurons are located only in the telencephalon forming a vast associational network that reciprocally interconnects limbic, allocortical, and isocortical structures (Frederickson and Moncrieff, 1994, reviewed in Frederickson et al., 2000). Zinc ions are potent modulators of glutamate receptors, especially the NMDA-mediated calcium influx, since the co-release of zinc along with glutamate provides a modulatory mechanism for postsynaptic excitability (Frederickson and Moncrieff, 1994; Calderone et al., 2004; Medvedeva et al., 2017). It is assumed that vesicular zinc at glutamatergic cells (mainly pyramidal-like and pyramidal cells) in the amygdaloid complex, hippocampus, and the perirhinal region is involved in the generation of synaptic plasticity related to neurodevelopment and learning or seizures in TLE (Frederickson and Moncrieff, 1994). Interestingly, the major glutamatergic fibers within the brain stem, thalamus, and cerebellum do not involve pyramidal cells and lack vesicular zinc (Frederickson and Moncrieff, 1994). Although not all pyramidal cells are zinc-containing neurons, the presence of this cell type in an environment with a potent modulation of glutamate receptors, calcium influx, and chances for hyperexcitability would contribute to epileptogenesis in TLE. Moreover, the synchronous firing of thousands of neurons repetitively for many seconds or more during a seizure, with intense release of glutamate and zinc, can induce marked postsynaptic calcium influx and synaptic remodeling. Indeed, patients with refractory TLE and comparable animal models of focal epilepsy have consistently reported a marked decrease in dendritic spine density on hippocampal and neocortical pyramidal cells (Swann et al., 2000). The loss of branches and the occurrence of varicose swellings on the remaining dendrites may alter local electrical signaling and contribute to epileptogenesis and clinical manifestations (Jiang et al., 1998).

Pyramidal cells are crucial but not the only neurons participating in seizure activity. GABAergic interneurons have been implicated in different aspects of seizure formation, contributing to the transition to ictal events through rebound excitation (Chang et al., 2018) and increasing the seizures/ictal activity duration (Khoshkhoo et al., 2017; Cela and Sjöström, 2019). GABAergic inhibition controls excitatory feedback (Naumann and Sprekeler, 2020). That is, the enhanced inhibition of inhibitory interneurons may result in the disinhibition of pyramidal cells with a consequent abnormal increased synchrony in the output of the hippocampus (Wittner et al., 2002). Interestingly, excitatory synapses between pyramidal neurons to subsets of interneuron types in hippocampus and neocortex expressed calcium-permeable AMPA-type glutamate receptors modulating the synaptic dynamics in local circuits (Lalanne et al., 2018). On the other hand, an increased perisomatic inhibition onto CA1 pyramidal cells can contribute to the generation and maintenance of abnormal synchrony in this region during hyperexcitability, interictal spikes, and epileptic seizures in humans with TLE (Wittner et al., 2005; Wittner and Maglóczky, 2017). GABAergic interneurons can also have an excitatory effect, by depolarizing the cells, and building a ‘positive feedback circuit’ together with glutamatergic pyramidal cells (Khazipov, 2016). In conjunction, these circuits’ rearrangements can lead to neuronal synchronization and hyperexcitability in cortical areas (Fujiwara-Tsukamoto et al., 2004).

## Integrating Pyramidal Morphology on Complex Networks in the Human Brain

To detail the nerve cells and their functional organization in multiple circuits is crucial to understand the human brain (Szentágothai, 1978; Ramón y Cajal, 1909–1911; Amaral and Insausti, 1990; Larriva-Sahd, 2002; Elston, 2003; DeFelipe, 2011; Geschwind and Rakic, 2013; Larriva-Sahd, 2014; Glasser et al., 2016; Luebke, 2017; Aru et al., 2019; Zeng, 2020). The neural circuits’ architecture dynamically combines the transmission, processing, and integration of information across cellular domains along space and time (Gertler et al., 2008; Spruston et al., 2013). The evolved elaboration and diversity of neural functions and the experience-dependent plasticity amend both discrete and continuous structural heterogeneity of cell types (Ramón y Cajal, 1894b; Holtmaat and Svoboda, 2009; Geschwind and Rakic, 2013; Cembrowski and Spruston, 2019), their dendritic geometry (Luebke, 2017), spine features (Yuste, 2013; Dall’Oglio et al., 2015), and axonal architecture (Beyeler et al., 2018; Rockland, 2020). These morphological aspects can differentiate neurons between species and their functions (DeFelipe, 2011). The pattern of synaptic organization of different pyramidal neurons is elaborated for each cortical area in the human brain, at the same time that is set at the level of each dendritic segment and the modulatory processes made by each spine (Andersen et al., 2007; Bourne and Harris, 2009; Chen and Sabatini, 2012; Spruston et al., 2013; Brusco et al., 2014; Chen et al., 2014; Scholtens et al., 2014; Stewart et al., 2014; Dall’Oglio et al., 2015; Hayashi-Takagi et al., 2015; Palomero-Gallagher and Zilles, 2017; Radler et al., 2020).

The morphological heterogeneity of pyramidal neurons can be observed along the subcortical-allocortex-neocortex *continuum* in the human brain. Pyramidal neurons have spines of all shapes, sizes, and likely functional properties. Indeed, the aspect of the long spiny CA1 hippocampal neuron shown here is impressive (Figure 9). The implicit functional complexity related to the huge density and variety of spine shapes along hundreds of dendritic micrometers of this pyramidal neuron would serve to merge the simple architecture of the hippocampus with its diversity of associated functions (Cembrowski and Spruston, 2019). Morphological differences relate to electrophysiological and functional implications for hippocampal pyramidal neurons in the deep and the superficial CA1 sublayers in the rat (Mizuseki et al., 2011). We also wonder how many of these human spines would be stable or arise as a plastic response to initiate the cascade of intracellular events for memory formation? Afterward, how many spines would disappear to let the dendritic membrane available for new synapses in a process that has to occur along with decades of the human lifespan. This “within-cell-type heterogeneity may provide the hippocampus the intrinsic flexibility that is needed to meet the diverse and variable demands of the external world” (Cembrowski and Spruston, 2019), as well as our internal states, personal memories and identity, and resilience responses (Rasia-Filho et al., 2018 and references therein).

Consistent with the previous proposition of Heimer et al. (2008), specific nuclei in the amygdaloid complex can be at the beginning of the limbic lobe, merging subcortical and allocortical parts in the basal forebrain. Specific amygdaloid nuclei display pyramidal-like and pyramidal neurons. The geometry of each neuron would be adapted to the nuclear area, the course of afferent pathways, and the local, intrinsic connectional organization. These are likely explanations for specific pyramidal-like neurons display apical dendrites oriented to other spatial directions than the pial surface, when present in transition areas prior to a clear cortical laminar organization. The CA3 and CA1 pyramidal neurons have dendrites with a spatial orientation and functional characteristics related to the hippocampal laminar connectivity. This morphological and functional coupling also occurs for the cytoarchitecture of the neocortical cells in the human brain. The pattern and distribution of inputs amend the dendritic architecture of neurons with distinct basal and apical domains adapted for each tissue volume (Spruston, 2008; Larriva-Sahd, 2014; Cembrowski and Spruston, 2019). It is challenging to consider the degree of normal variability that is possible to occur for these cells and circuits for every one of us. For example, the gray matter volume in the cortical areas related to complex visual-spatial, auditory, motor skills, connectivity of the tracts, and the functional activation overlap of language and music are all significantly larger in musicians than in non-musicians (Gaser and Schlaug, 2003; Bouhali et al., 2020). This is in line with the Ramón y Cajal’s (1894a) statement: “it can be admitted as very probable that mental exercise leads to a greater development of the dendritic apparatus and of the system of axonal collaterals in the most utilized cerebral regions. In this way, associations already established among certain groups of cells would be notably reinforced by means of the multiplication of the small terminal branches of the dendritic appendages and axonal collaterals.” Moreover, “if a given classical cell type actually embodies a collection of heterogeneous elements, such a cell type could perform a corresponding collection of operations. In the case of cell types that repeat across space, such within-cell-type heterogeneity could facilitate the simultaneous execution of distinct computations through the same apparent circuitry” (Cembrowski and Spruston, 2019).

Going one step further, pyramidal neurons increased their receptive surface and modulatory capabilities by having multiple shaped spines for synaptic processing, compartmentalization, stability, or plasticity with impact on the shaft dendrite and vice-versa (Harris, 1999; Bourne and Harris, 2009; Yuste, 2013). Dendrites operate with linear, sublinear, and supralinear summation of frequency, amplitude, and time window of postsynaptic responses (Tran-Van-Minh et al., 2015). Pre- and postsynaptic plasticity show complementary functions (Mizusaki et al., 2018). The presynaptic element adjusts the speed of learning and controls over postsynaptic firing rates whereas postsynaptic plasticity regulates spike timing and frequency and amplifies the response range (Mizusaki et al., 2018). Another important feature is that spines activated by specific stimuli are found widely distributed on basal and apical dendrites (Chen X. et al., 2011). That is, spines responsive to auditory stimuli are found interspersed on the same dendritic branch, and adjacent spines can respond to different sound frequencies in the mouse cerebral cortex *in vivo* (Chen X. et al., 2011). When synaptic wiring is random, the dimension of a representation formed by many sparsely connected neurons can be higher than that of a smaller number of densely connected elements (Litwin-Kumar et al., 2017). If this model could be applied similarly to dendritic spines, it would be possible that multiple connections in different spines along the pyramidal dendrites would provide a high-dimensional representation and output pattern to different ensembles of inputs (adapting data from Litwin-Kumar et al., 2017). On the other hand, it is possible that both the scattered and the clustered synaptic processing can be relevant for the dendritic integration strategies and to determine which prominent dendritic mechanism will be recruited for postsynaptic summation (Tran-Van-Minh et al., 2015). “The recruitment of synapses that participate in the encoding and expression of memory is neither random nor uniform… The clustering of synapses may emerge from synapses receiving similar input, or via many processes that allow for crosstalk between nearby synapses within a dendritic branch, leading to cooperative plasticity. Clustered synapses can act in concert to maximally exploit the non-linear integration potential of the dendritic branches in which they reside. Their main contribution is to facilitate the induction of dendritic spikes and dendritic plateau potentials, which provide advanced computational and memory-related capabilities to dendrites and single neurons” (Kastellakis and Poirazi, 2019). In conjunction, these findings indicate that the diversity of operations in pyramidal spines provide an exceptional repertoire for the integration of postsynaptic potentials at each spiny dendritic segment and more “functional output codes” for each cell.

Notably, human pyramidal neurons are not merely “scaled-up” versions of neurons found in other species but have improved structural and encoding capabilities properties (Mohan et al., 2015). Single-cell RNA-sequencing datasets revealed particularities in gene expression, morphology, proportions, and laminar distributions of cell types in our cerebral cortex (Hodge et al., 2019). There are also species-specific differences in key molecules that regulate synaptic plasticity (Beed et al., 2020). Human CA1 pyramidal neurons have larger apical and basal dendrites with higher branching complexity than mice (Benavides-Piccione et al., 2020). Human pyramidal neurons in layers II/III of the temporal cortex have threefold larger dendritic length and increased branch complexity with longer segments than in macaque and mouse (Mohan et al., 2015). *In silico*, human pyramidal cells with larger dendritic trees track the activity of synaptic inputs with higher temporal precision, enabling efficient information transfer from inputs to output within cortical neurons (Goriounova et al., 2019). Coexisting synapses on dendritic shafts and axo-spiny contacts at different distances from the soma in the dendritic arbor influence the neuron’s excitatory and inhibitory integrative capacity (Megías et al., 2001; Kubota et al., 2007; Anderson et al., 2009; Spruston et al., 2013; Bucher et al., 2020). There is also a high level of interdependence between dendritic excitability and synaptic plasticity, i.e., activity-dependent regulation of dendritic excitability induces synaptic plasticity, and synaptic plasticity affects dendritic computations (see Ramaswamy and Markram, 2015). Spines add more plasticity to synaptic transmission, serving as time-space encoding and decoding devices, and involving varied number and intermingled shapes and sizes for a moment-to-moment activity and engrams. Human dendritic spines have neck length about 30% longer and 100% more volume than in the somatosensory cortex of mice (DeFelipe, 2011 and references therein). The monkey prefrontal cortex layer III pyramidal neurons have spatially (non-random) clustered dendritic spines (mushroom-like and stubby ones) predominantly concentrated in apical terminal branches (Yadav et al., 2012). The same pattern was not found in basal and apical dendritic segments of layer III pyramidal neurons from frontal, temporal, and cingulate cortex in humans (two males; Morales et al., 2014).

Let us then consider that the morphological heterogeneity of human pyramidal neurons also implies likely differences in functional properties. There is a high degree of synaptic diversity arising from molecular and morphological differences among individual synapses and spatially distributed within individual dendrites, between different neurons, and across and between brain regions, which can produce a non-uniform spatial output of synaptic potentials (Grant and Fransén, 2020). The intra-individual and inter-individual differences associated with the potential structural plasticity of the pyramidal dendritic spines imply much more probabilistic possibilities for the functional organization of the human cortical areas. How would all these pyramidal features in each specific area encode, integrate, and determine the conscious identity of each of us? There is no complete explanation for this question yet. Adding to the variations of the shape and connectivity of pyramidal neurons in different human brain areas (Jacobs et al., 1997, 2001), this scenario is enriched by: (1) the stereological estimation of 12.2 million neurons in the amygdaloid complex (approximately 8.5 million in the basolateral nuclei; Schumann and Amaral, 2005); (2) 2.7 million pyramidal neurons in CA2-3 and 16 million pyramidal neurons in CA1 (West and Gundersen, 1990); (3) 16 billion cortical neurons (Herculano-Houzel et al., 2014); and (4) 5 billion neurons in the temporal lobe of humans (Pakkenberg and Gundersen, 1997; see von Bartheld et al., 2016 for a review). When estimating the number of neurons and of the synaptic profiles within cubes of cortical tissue (50 μm wide by 50 μm thick) in the layer V of the human anterolateral temporal cortex, there are 21 neurons and 958,890 synapses, which gives approximately 30,000 synapses per neuron, 90% being asymmetric and 10% symmetric ones (DeFelipe, 2011). Multi-sites non-linear signals and large excitatory synapses/cell (∼30,000) can enhance the computational capabilities for the comparatively short human temporal layer II/III pyramidal neurons (Eyal et al., 2018). Pyramidal neurons in the prefrontal cortex can have up to 23 times more dendritic spines than those in the primary visual area (Elston, 2003). Moreover, human temporal pyramidal neurons possess unique biophysical membrane properties that significantly enhances both synaptic charge-transfer from dendrites to soma and spike propagation along the axon (Eyal et al., 2016). The axon hillock location relative to the soma or dendrite is finely tuned with the somatodendritic capacitive load in thick-tufted pyramidal neuron in neocortical layer V (Hamada et al., 2016). Finally, by miniaturization of computational gating, it was calculated that the human cerebral cortex executes over 1.2 zetta logical operations per second without combusting the brain by the released heat (Georgiev et al., 2020).

We focused our work on pyramidal neurons, but we should not dismiss other important issues as: (1) the interneurons needed for the cortical functioning (the “neurons with short axon” described by Ramón y Cajal, 1909–1911; Fairén et al., 1984; McCormick et al., 1993; Gabbott et al., 1997; Kubota et al., 2007; Larriva-Sahd, 2014; Jiang et al., 2015; Ramaswamy and Markram, 2015; Gouwens et al., 2019); (2) the whole-genome and transcriptome studies for cell origin and evolution and, more specifically, for the heterogeneous pyramidal neurons across the cortical areas (Hill and Walsh, 2005; Thompson et al., 2008; Geschwind and Rakic, 2013; Arendt et al., 2016; Cembrowski and Spruston, 2019; Writing Committee for the Attention-Deficit/Hyperactivity Disorder, et al., 2020); (3) the spine structure modulation by microRNA epigenetic actions (Park et al., 2019) or by other neurotransmitters than glutamate (e.g., dopamine; Yagishita et al., 2014; Iino et al., 2020); and (4) the contiguous glia with regional functional and morphological specializations (Hodge et al., 2019), plasticity, heterogeneity (Chai et al., 2017), cooperativity for synaptic communication, remodeling, and integration with axons, dendrites, spines, and the extracellular matrix (Dityatev and Rusakov, 2011; Stewart et al., 2014; Chai et al., 2017; Mederos et al., 2018; Arizono et al., 2020; Chioma et al., 2020).

We have shown images from males. Nevertheless, pyramidal neurons in the rat CA1 hippocampal region are subject to various modulatory factors that can affect spine number and shape depending on sex steroids, such as fluctuations in estrogen and progesterone circulating levels and the expression of aromatase (Woolley et al., 1990; Brusco et al., 2008; Yague et al., 2010; Hansberg-Pastor et al., 2015; Sheppard et al., 2019; Barreto-Cordero et al., 2020). Indeed, morphological differences related to sex were reported for human insular pyramidal neurons (Anderson et al., 2009) and for the hippocampal estrogen receptor-α localization in neurofibrillary tangles along with AD (Wang et al., 2016). The modulatory effects of gonadal hormones on human neuronal and glial structure and circuits are another avenue open to further research (e.g., Cahill, 2006; Gobinath et al., 2017; Fogazzi et al., 2020).

## Concluding Remarks

Here, we discussed the emergence and heterogeneity of pyramidal neurons, as well as the dendritic spine diversity in specific amygdaloid nuclei at the beginning of the limbic lobe, progressing along with allocortical and neocortical areas in the human brain. Additional morphological and functional contributions to this field are welcome and can employ and/or expand the present 3D reconstruction procedure in other human brain areas. The involvement of pyramidal neurons (Pierri et al., 2003; Petanjek et al., 2019) and dendritic spines in normal development or in neurological and psychiatric dysfunctions is an important ongoing research field (Ferrer et al., 1986; Ferrer and Gullotta, 1990; Fiala et al., 2002; Blazquez-Llorca et al., 2011; Merino-Serrais et al., 2011; Penzes et al., 2011; Dorostkar et al., 2015; Ramaswamy and Markram, 2015; Herms and Dorostkar, 2016; Chioma et al., 2020). The use of computational tools to explore structural and functional relations of human pyramidal neurons (Toharia et al., 2016) with a model-based clustering mathematical approach for dendritic spines can add theoretical predictions on the functional features of human pyramidal neurons and their integrated synaptic processing (Luengo-Sanchez et al., 2018). We would like to contribute with additional information on the morphological heterogeneity observed in human pyramidal neurons and spines, relevant to elucidate much of the neural processing across various parts of the human brain and in comparative studies with other species (DeFelipe, 2011; Geschwind and Rakic, 2013; Soltesz and Losonczy, 2018 Cembrowski and Spruston, 2019).

## Data Availability Statement

All data are available as shown in the present report. Additional data can be requested directly from the authors.

## Ethics Statement

The studies involving human participants were reviewed and approved by The Brazilian Ethics Committee from the Federal University of Health Sciences of Porto Alegre (UFCSPA; #43059115.4.0000.5345, #48771715.5.0000.5345, #06273619.7.0000.5345, and #18718719.7.0000.5345) and Universidade Federal do Rio Grande do Sul (UFRGS; #18718719.7.3001.5347). The next of kin provided written informed consent for brain donation and for use in this kind of study. There is no potentially identifiable data for any individual included in this article.

## Author Contributions

AR-F, KG, CV, AD, RR, CJ, and MC: study concept and design. AR-F, CV, and AD: acquisition of data. AR-F, KG, CV, and AD: two-dimensional reconstructions. AR-F, KG, RR, and CJ: three-dimensional reconstructions. AR-F, KG, CV, AD, RR, CJ, and MC: interpretation of data and elaboration of the manuscript. All authors contributed to the article and approved the submitted version.

## Conflict of Interest

The authors declare that the research was conducted in the absence of any commercial or financial relationships that could be construed as a potential conflict of interest.

## Supplementary Material

Supplementary Figure 1 Three-dimensional reconstruction of a Golgi-impregnated pyramidal-like neuron from the human cortical amygdaloid nucleus.

Supplementary Figure 2 Three-dimensional reconstruction of a Golgi-impregnated pyramidal-like neuron from the human basomedial amygdaloid nucleus.

Supplementary Figure 3 Three-dimensional reconstruction of a Golgi-impregnated pyramidal neuron from the human basomedial amygdaloid nucleus.

Supplementary Figure 4 Three-dimensional reconstruction of a Golgi-impregnated pyramidal neuron from the human CA3 hippocampal region.

Supplementary Figure 5 Three-dimensional reconstruction of a Golgi-impregnated small pyramidal neuron from the human CA1 hippocampal region.

Supplementary Figure 6 Three-dimensional reconstruction of a Golgi-impregnated large pyramidal neuron from the human CA1 hippocampal region.

Supplementary Figure 7 Three-dimensional reconstruction of a Golgi-impregnated small pyramidal neuron from the human external layers II/III in the anterolateral temporal neocortex.

Supplementary Figure 8 Three-dimensional reconstruction of a Golgi-impregnated large pyramidal neuron from the human internal layer V in the anterolateral temporal neocortex.
